# Integrating Monitoring and Biomonitoring Data with Mechanistic Models to Better Estimate and Characterize Aggregate Human Exposures to Semivolatile Organic Chemicals

**DOI:** 10.1021/acs.est.5c08964

**Published:** 2025-12-02

**Authors:** Lauren Hughes, Jirka Cops, Lieve Geerts, Katleen De Brouwere, Alessandro Sangion, Li Li, Jon A. Arnot

**Affiliations:** † ARC Arnot Research and Consulting Inc., 393 Ashdale Ave., Toronto, Ontario M4L 2Z3, Canada; ‡ Flemish Institute for Technological Research (VITO), Boeretang 200, 2400 Mol, Belgium; § School of Public Health, 6851 University of Nevada Reno, Reno, Nevada 89557-274, United States; ∥ Department of Pharmacology and Toxicology, University of Toronto, Medical Sciences Building, 1 King’s College Circle, Toronto, Ontario, Canada M5S 3K3; ⊥ Department of Physical and Environmental Sciences, University of Toronto Scarborough, 1065 Military Trail, Toronto, Ontario, Canada M1C 1A4

**Keywords:** indoor environments, aggregate human exposure, monitoring data, human biomonitoring data, mass balance models

## Abstract

Humans are exposed to many chemicals from multiple sources through various pathways. Many semivolatile organic chemicals (SVOCs) are ubiquitous in indoor environments, but the extent of exposure and relative importance of different pathways (near-field or far-field) are uncertain. Here, 37 SVOCs with measured concentrations in indoor media are used in conjunction with a mass balance indoor fate, exposure, and toxicokinetic model to 1) estimate exposures from indoor environments, 2) incorporate measured dietary (far-field) exposures, 3) evaluate modeled biological concentrations in blood and urine against previously published human biomonitoring (HBM) estimates, 4) calculate the relative importance of different exposure pathways, and 5) demonstrate the value of using models and monitoring data to estimate aggregate exposure for human health assessment. All model calculated blood and urine concentrations are within 2 orders of magnitude of HBM values, and 73% are within 1 order of magnitude. The method explicitly considers uncertainty in measured indoor concentrations and mouthing-mediated ingestion (MMI). When median measured chemical concentrations in dust and MMI are used for modeling, far-field dietary intake is determined to be the dominant contributor to the overall exposure for almost all investigated SVOCs. However, when modeling with higher (third quartile reported) measured chemical concentrations in dust and ∼ 3x higher dust ingestion rates, near-field sources result in exposures for some SVOCs that are comparable to or exceed contributions from far-field exposure pathways. The model also addresses measurement data gaps and, combined with the monitoring data, provides a method to estimate chemical emission rates.

## Graphical abstract

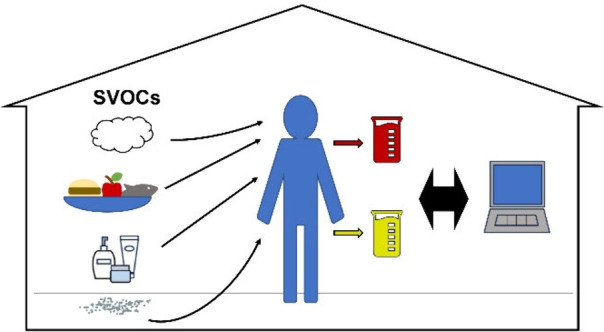

## Introduction

Chemicals are being evaluated for exposures and potential risks to humans;^1–4^ however, measured exposure data sets are usually incomplete even for relatively well-studied chemicals. Uncertainty in exposure data is abundant including crucially important chemical use and emission rate data.^5–7^ A vision for exposure science in the 21st century^8^ includes an exposure narrative in which quantitative relationships between chemical sources, fate and transport processes, external exposure, and internal exposure are established (an “exposure-tracking framework”) to better inform decisions on human and ecosystem health. Near-field human exposures include indirect (chemical release to the immediate indoor environment) and direct (to the body) sources, while far-field exposures to “humans via the environment” include inhalation of outdoor air and ingestion of food and drinking water.^9,10^ If complete measurements for chemicals in air, drinking water, food, dust, personal care products (PCPs), and other consumer products are available, these multimedia concentrations can be coupled with human exposure factors to calculate *exposure rates*, e.g., mg-chemical/kg-body weight/day. However, such complete data sets are rare, and exposure rate calculations do not include toxicokinetic processes for determining systemic exposures near target sites. On the other hand, human biomonitoring data (HBM) from blood and urine samples provide excellent snapshots of internal exposure *concentrations*, e.g., μg/L. While HBM data provide aggregate exposure information they cannot determine the sources of exposure. A suitable holistic quantitative exposure assessment framework should integrate all sources, pathways, and routes of exposure as well as toxicokinetic models for calculating internal exposures from external exposure rates; the agreement between the calculated internal exposures and HBM can then provide confidence in the exposure estimates. When measured data are absent or incomplete, mechanistic (process-driven) mass balance models can be used to estimate route-specific and aggregate external and internal exposures relative to emissions or from chemical production volumes.^11^

Many semivolatile organic chemicals (SVOCs), such as plasticizers, pesticides, brominated flame retardants (BFRs), organophosphate esters (OPEs), and short- and medium-chain chlorinated paraffins (SCCPs and MCCPs), are used and dispersed broadly throughout manufactured and natural environments. Indoor sources include construction materials, electronic equipment, furniture, insecticides, plastics, PCPs, and other consumer products. SVOCs can be released from these sources to the indoor environment by volatilization, abrasion, or degradation. Pesticides can be emitted indoors by direct spray application. Indirect near-field human exposure to SVOCs occurs through inhalation, dermal absorption from skin contact with surfaces or air, and mouthing-mediated ingestion (MMI) of dust or surface residues (i.e., hand-to-mouth or object-to-mouth).^12^ Humans can also be directly exposed through all routes (ingestion, inhalation, and dermal) to SVOCs in pharmaceuticals and PCPs. Many SVOCs are also quantified in far-field exposure sources, e.g., food. Because many SVOCs are multimedia contaminants quantifiable in indoor and outdoor air, dust, and food, aggregate human exposure estimation requires a significant amount of information. Given the complexity of human exposures to SVOCs, interpreting HBM data to better understand dominant sources and pathways, including possible risk mitigation, is challenging. For example, the relative contribution of near- and far-field sources of SVOCs to overall exposure is not always clear.^13–15^ Some research suggests dust ingestion can be the primary route of exposure to SVOCs used indoors^13,16–19^ while other studies indicate that far-field dietary exposures play a more significant role than near-field sources in human exposure to recalcitrant SVOCs released exclusively indoors.^15^ Exposure is dependent on the chemical properties, uses, and source strengths. Rigorous chemical evaluations should consider multiple sources, pathways, and routes in a consolidated manner to obtain aggregate exposure estimates.^20,21^ The Risk Assessment IDentification And Ranking – Indoor and Consumer Exposure (RAIDAR-ICE) model^22^ combines an indoor multimedia mass balance chemical fate module with a human exposure and physiologically based biokinetic (PBK) module to calculate external and internal exposures to chemicals through multiple near-field direct and indirect exposure routes (e.g., inhalation, ingestion, and dermal exposure). RAIDAR-ICE can also include estimated or measured exposures from far-field sources, e.g., chemical contamination in food. The PBK model calculates chemical concentrations in blood and urine, providing biologically relevant doses proximal to putative target sites, and facilitates exposure model evaluations with HBM data. RAIDAR-ICE is part of the Exposure And Safety Estimation (EAS-E) Suite framework (www.eas-e-suite.com) which includes not only an extensive database of chemicals and their properties (e.g., measured and calculated partition coefficients, degradation half-lives, toxicokinetic data) but also a module that can be used to calculate many of these properties from the chemical structure, i.e., Simplified Molecular Input Line Entry System (SMILES) notation, when such data are not otherwise available.^23^

The primary objective here is to demonstrate how a mass balance modeling framework can be used to integrate limited monitoring data (pieces of exposure information) to estimate aggregate exposure intake rates and biological concentrations, providing a more complete exposure assessment in which various assumptions can be examined, available measured data scrutinized, and sources and pathways of measured HBM data better understood. In this study, the RAIDAR-ICE model is parametrized with the required chemical property parameters (e.g., partition ratios, half-lives) for 37 neutral SVOCs, whose concentrations in air and dust are reported from the “Screening of cHemicals in the Indoor enviroNment for human Exposure assessment (SHINE)” project.^24–26^ Specifically, to address a key source of uncertainty for parametrizing fate and exposure models, we use the indoor air and dust monitoring data and the model to “back-calculate” plausible emission rates.^27,28^ The emission rate estimates calculated from measured concentrations in one medium (vacuumed dust or indoor air) are used to calculate chemical fate and concentrations in all remaining media in the modeled indoor environment as well as subsequent intake and uptake rates. The model then combines the chemical and route-specific near-field intake rates with dietary intake rates and dermal application rates (for three chemicals for which such rate data are available^29^) collected from the literature to calculate aggregate exposure estimates and biological concentrations for comparison to independent HBM data reported in studies from European populations. The exposure pathways of inhalation of indoor air, dermal absorption, and dietary and dust ingestion were explicitly quantified and compared providing key insights into human exposure to these chemicals. The mechanistic MMI model was used to explore the uncertainty in chemical concentrations in dust and hand-to-mouth contact.

## Materials and Methods

Figure 1 provides a conceptual overview of the methods that combine available monitoring data with the RAIDAR-ICE model to calculate aggregate exposure estimates and compare the modeled human exposures with independent HBM data. The steps to this process are summarized below. Concentrations of SVOCs from outdoor air were excluded from far-field modeling, because such concentrations are typically lower than those found indoors^30,31^ and the modeled human in this study is assumed to spend 90% of their time indoors.

**Figure 1:**
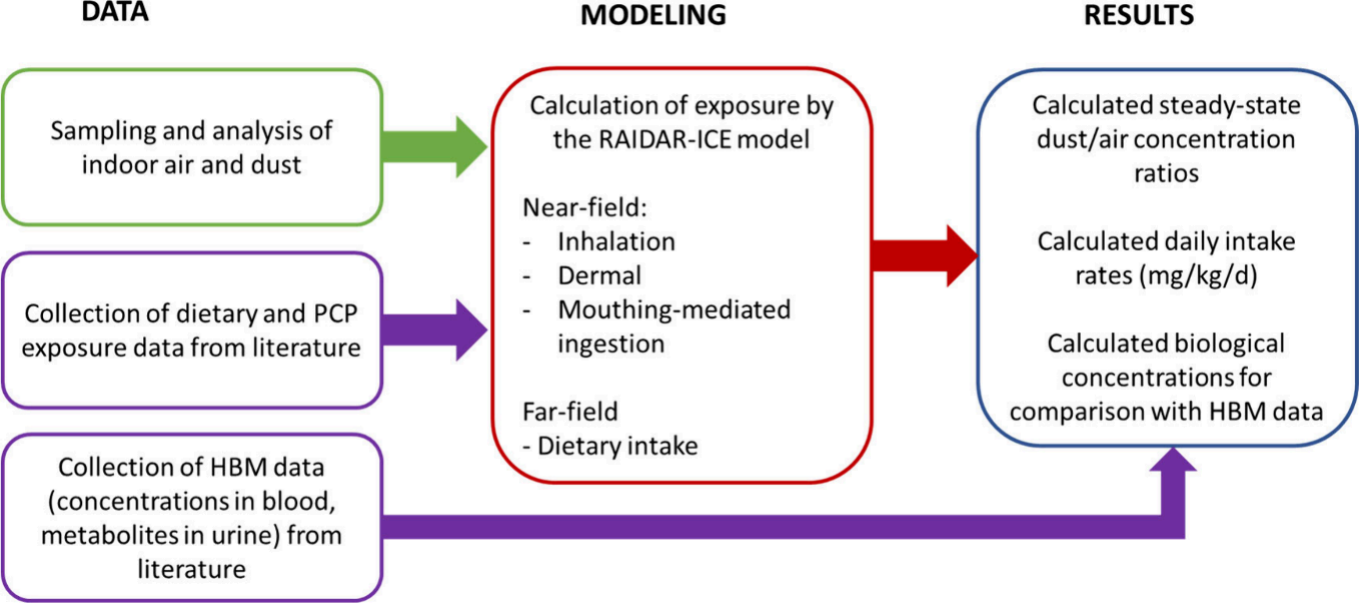
Schematic overview of the exposure routes and data considered in this work.

### Case Study Chemicals and Measured Air and Dust Concentration Data

Table S1 in the Supporting Information (SI) lists the case study chemical names, abbreviations, CAS numbers, and SMILES notations, and Table S2 summarizes their measurements in indoor air and floor dust from the SHINE project. The samples were gathered from various indoor settings (day care centers, offices, and homes) in Belgium, The Netherlands, Ireland, and Sweden over a period of six months between December 2016 and May 2017 to screen for a large number of both legacy and emerging SVOCs found indoors, and some of these data have been previously published.^24–26^ The 37 case study chemicals were selected for their high detection frequency and high levels in air or dust samples, their coverage of several chemical classes (e.g., plasticizers, pesticides, etc.), and the availability of HBM data from the European population. Data across all sampled regions and settings were combined to calculate median concentrations, which are considered generally representative of “average” contamination levels in an archetypal indoor environment and reflect the central tendency of human exposure. This was a necessary simplification because complete sets of region-specific data (e.g., concentrations in indoor media, dietary intake data, and HBM data from the same country) were not available for most chemicals. Also, despite inherent spatial and temporal heterogeneity, using these average levels does not introduce significant bias because for most chemicals the mean concentrations did not vary by more than 1 order of magnitude between locations. Samples reported below the limit of detection (LOD) were included in the calculation and assigned a value of the LOD multiplied by the detection frequency. Samples between the LOD and limit of quantification (LOQ) were assigned values of (LOD + LOQ)/2.

### Derivation of Dietary Intake and Dermal Application Data

Table S3 summarizes dietary intake data (central tendency estimates at the population level) collected from the literature with a focus on data from Northern Europe collected in the same period as SHINE sampling (2016–2017) when possible. Due to a lack of measured dietary data, a zero value was assumed for bis­(2-ethylhexyl) terephthalate (DEHT) and some brominated diphenyl ethers (BDEs-206, -207 and -208). Apart from the passive absorption of gaseous chemicals from indoor air and chemical residues on the skin surface, direct chemical application to the body is also important in determining human exposure to certain chemicals in PCPs, including some phthalates in this study. Therefore, we additionally include dermal application data for dimethyl phthalate (DMP), diethyl phthalate (DEP), and diisobutyl phthalate (DiBP). Table S4 provides a summary of the dermal product application data.^29^

### Human Biomonitoring Data

Table S5 summarizes the blood and urinary HBM data (central tendency estimates at the population level) collected from the literature with a focus on data from Northern Europe with high detection frequencies collected in the same period as SHINE sampling and from adults to the greatest extent possible. The data were collected from a range of ages and included both male and female subjects. HBM data were not based on samples from the occupants of the indoor spaces where concentrations in air and dust were measured. The HBM blood data were reported as ng/g lipid in serum and were converted to whole blood concentrations (μg/L) to compare with model calculations by assuming that serum concentrations are at equilibrium with plasma concentrations, that whole blood consists of 55% of plasma, and that the average total serum lipids in blood are 7.05 g/L.^32^

### Model Description and Parametrization

The RAIDAR-ICE model^22^ continues to evolve through iterative testing and refinement (see SI for model update notes) and the Ver.1.8 model was used in this study. The minimum set of chemical-specific property parameters required for model simulations are molar mass, octanol–water partition ratio (*K*_OW_), octanol–air partition ratio (*K*_OA_), degradation half-lives in air and on indoor surfaces, and whole-body biotransformation half-lives (HL_B_); it additionally allows integrating information on dietary intake rate (if far-field exposure is included in the simulation) and information on human dermal product application (if consumer dermal exposure is considered).^22^ Table S6 lists log K_OW_, log K_OA_, and reaction half-lives for the chemicals modeled in this work, retrieved from the EAS-E Suite chemical database unless otherwise indicated.

The default RAIDAR-ICE parametrization characterizes general indoor conditions and the physiology and behavior of an “average” adult.^22^ For instance, RAIDAR-ICE considers chemical distribution and transfer across various indoor compartments, including air, vinyl flooring, carpet, polyurethane foam furniture, and hard surfaces (tables, walls, ceiling etc.) The modeled individual possesses anthropometric and physiological properties of an 80 kg male adult. Every day, the male adult touches the carpet, vinyl flooring, polyurethane foam, and hard surfaces 12, 12, 24, and 24 times, respectively, and has 24 hand-to-mouth contact events.^12^ The male adult washes his hands three times and takes a bath each day. Based on the above contact frequencies and default dust loadings, the model calculates a default dust ingestion rate of 6.2 mg/day. Chemicals can also partition into settled dust (described as size bins from <0.1 to >150 μm) found on various indoor surfaces, where dust can be introduced through the track-in, emission, and deposition processes, resuspended into the indoor air, and removed through surface cleaning. RAIDAR-ICE employs an indoor particle mass balance module to calculate dust loadings on various indoor surfaces.

This modeling exercise is intended as a screening-level analysis focused on understanding chemical fate and exposure patterns under generic indoor conditions. It does not aim to replicate individual-level exposure scenarios or specific building environments. By abstracting from environmental heterogeneity, we aim to identify robust, system-level insights that can be transferred across a range of settings.

The RAIDAR-ICE model requires user-specified chemical emission rates (ng/h) to calculate concentrations in indoor media. Because RAIDAR-ICE is a linear steady-state model, the calculated chemical concentrations in the indoor and human compartments are proportional to the chemical emission rates. This linearity allows the “inverse” calculation of the emission rates that sustain the observed levels of chemical concentrations indoors.^15^ Emission rates to indoor air were estimated by fitting the predicted concentrations in dust (ng/g) or air (ng/m^3^) to the measured median concentrations. In brief, we first modeled concentrations in indoor media assuming a “unit emission” rate of 1 ng/h, generating reference values that represent the system’s response to a standard input. We then adjusted the unit emission rate upward or downward by a scaling factor to ensure that the calculated concentrations agree with the median concentrations measured in indoor environments from the SHINE project. The values after this adjustment are the emission rates presented in Table S2. Here, median concentrations measured in dust (ng/g) were used for all chemicals except for DMP; for DMP, the median concentration measured in air (ng/m^3^) was used because air had a much higher detection frequency compared to that of dust. Chemical emissions are assumed to occur only to indoor air. Back-calculated emissions are reported in units of ng/(m^2^ h) to account for differences in room size when these estimates are compared to other estimates. The dust samples were collected using vacuum cleaners from various types of flooring (e.g., vinyl, parquet, carpet etc.). Therefore, although RAIDAR-ICE calculated dust-bound chemical concentrations on individual indoor surfaces, its “concentrations in removed (e.g., vacuumed) dust”, which aggregates dust-bound chemical concentrations on floors and carpeting, was used to estimate emission rates. The dust removed by vacuuming accounts for the relatively higher removal efficiencies and lower organic carbon fractions of larger particles compared to smaller particles.^15^

The RAIDAR-ICE toxicokinetic model calculates parent chemical concentrations at a whole-body level and in blood and urine based on the calculated chemical intake rates. For persistent substances that are predominantly present in blood as their unchanged forms, i.e., BFRs and CPs, a direct comparison between model results and measured HBM data was feasible. However, for substances subject to rapid biotransformation in humans, i.e., pesticides, plasticizers, and OPEs, the comparisons between model results and measured HBM data were less straightforward since the HBM data sets report only the concentrations of metabolites. For example, mono­(2-ethylhexyl) phthalate (MEHP) is the main metabolite of di­(2-ethylhexyl) phthalate (DEHP). Therefore, we calculated metabolite concentrations in urine from the calculated parent chemical daily intake rate using the Fraction Urinary Excretion (*F*_UE_) method^33,34^ with experimentally observed *F*_UE_ factors retrieved from the literature as summarized in the SI. For example, DEHP has a *F*_UE_ factor of 0.059 for its main metabolite MEHP (Table S7), meaning that an intake rate of 1 mol/kg-bw/d of DEHP results in the excretion of 0.059 mol/kg-bw/d of MEHP through urination. Since permethrin and cypermethrin have three common metabolites (cis-DCCA, trans-DCCA and 3-PBA), modeled permethrin and cypermethrin exposure rates were summed for comparison with HBM data, as the metabolites could not be attributed to either one or the other.

## Results and Discussion

### Estimated Emission Rates

Table S2 shows the back-calculated emission rates to air normalized to room area (ng/(m^2^ h)) for the 37 case study chemicals span 6 orders of magnitude ranging from 1.8 × 10^–4^ (2,4-dibromo-1-(4-bromophenoxy)­benzene, BDE-28) to 78 (DEP). The back-calculated emission rates to air derived here with data from the EU were compared with previous estimates derived from measured dust and hand-wipe concentrations collected in the U.S. Table S9 and Figure S1 show that for the 24 SVOCs common to both studies, 16 (67%) of the emission rate estimates are within 1 order of magnitude. There is a strong positive correlation between the log-transformations of the two data sets (r^2^ = 0.83), and the intercept indicates that on average the emission rates are about 10× lower in the EU data compared to the US data. The largest differences are for less brominated PBDEs (28, 47, 49, 99, 100, 153, and 183) in which emission estimates from the US are about 40× higher. This difference may reflect the slight difference in time-frames for banning these chemicals in products and/or the quantities of such products in different indoor environments. Another notable difference is for DiBP in which estimated emission rates from the EU data were about 70× higher. A satisfactory explanation for this discrepancy could not be determined. Table S9 also includes previously published emission estimates from various materials (e.g., flooring, wallpaper, latex paint) and objects (e.g., printed circuit boards from computers) used indoors, calculated from chamber experiments and emissions modeling.^35–38^ A key difference between these data sources for emissions is that the back-calculated values are from various potential sources in an indoor environment and not a single new product or material in a controlled environment. There is merit in considering various methods to address uncertainty in chemical emission rates.

In the SHINE project, office characteristics and contents were not significantly correlated to indoor FR concentrations,^26^ and vinyl flooring was the only product significantly correlated with higher plasticizer concentrations in dust.^24^ Estimates of emission rates for DEHP from vinyl flooring derived from a combination of mechanistic modeling and chamber experiments^36,39,40^ range from 10^2^ to 10^3^ ng/(m^2^ h). Vinyl flooring makes up 16.3% of the total floor area in our modeled environment, leading to an expected total room emission rate of DEHP between 16 and 160 ng/(m^2^ h) from vinyl flooring alone. The rate of 72 ng/(m^2^ h) for DEHP back-calculated here from measured dust concentrations is in good agreement with the aforementioned estimate, but is much lower than what was reported in an earlier chamber experiment study.^35^ Vinyl flooring was not universal in the rooms sampled for the SHINE project, and emissions from flooring may depend on many factors including the initial concentration in the vinyl as well as age and wearing of the flooring over time.

Emission rates are largely unknown and variable depending on the quantities, types, and use scenarios for products, articles and building materials;^39^ therefore, the estimates calculated here can only be approximations at the order of magnitude level. Nonetheless, given the critical need for source and emission rate data, there is merit in obtaining emission rate estimates from various methods including inverse modeling and controlled experiments. These data can be used to test and validate “cradle-to-grave” model frameworks like PROduction-To-EXposure (PROTEX) that aim to calculate emissions and exposure from chemical production volumes and use data.^11^ Further efforts to better quantify emission rates from different methods are encouraged so that databases of context-specific chemical emission rates can be developed.

### SVOC Concentrations in Air and Dust

Figure S2 compares model calculated dust–air concentration ratios (in m^3^/g) with measured dust–air concentration ratios sampled from various indoor environments for 26 case study chemicals. Modeled dust–air concentration ratios were within 1 order of magnitude of the median measured values for 21 of the 26 SVOCs and within 2 orders of magnitude for all chemicals but decabromodiphenyl ethane (DBDPE; measured 10^1.75^ m^3^/g vs modeled 10^3.9^ m^3^/g). The measured concentrations in air and dust were not derived from simultaneous, paired measurements in the same indoor space, which are likely to bias certain samples and therefore contribute to some of the discrepancy between modeled and measured ratios. For example, for DBDPE the measured chemical concentrations in dust used to estimate emission rates were collected from several settings in four different countries, whereas air sampling for DBDPE was carried out in only Swedish offices and Irish homes. Concentrations in both media in Ireland were 2 or more orders of magnitude higher than measured elsewhere, consistent with previously reported data.^13^ The smaller sample pool of air data skews the median concentration in air higher (due to the high values from Ireland), while the larger pool of dust data from other countries yields a lower median concentration in dust. The dust–air concentration ratio for DBDPE calculated using median measured values from Ireland exclusively is 10^2.5^, still lower than the value of 10^3.9^ calculated by the model but in better agreement than the value of 10^1.75^ calculated from the whole data set (Figure S2). The lower values are unexpected given DBDPE’s high log K_OA_ value (18.8), and it is reasonable to hypothesize that its dust–air concentration ratio would be closer to that of BDE-209 (10^4.9^ measured, 10^3.9^ modeled). The model results also appear to overpredict air concentrations for α-hexabromocyclododecane (α-HBCDD) and BDE-206; however, these chemicals had very low detection frequencies (less than 40%) in air which resulted in their calculated median concentrations being lower than the reported LODs.^26^ The subsequent dust/air concentration ratios are therefore biased in their estimation. Similarly, median concentrations of DMP in air and dust included many nondetects and should therefore be interpreted cautiously.

Measured concentration ratios from paired dust and air samples in 10 different Swedish offices show variability of a factor of 30 or less for all but two OPEs and BFRs.^26^ Note that the dust–air concentration ratios in this study can be different from (equilibrium) dust–air partition coefficients measured in laboratory conditions. The dust–air concentration ratio does not assume unlimited length of contact between dust and air, as required by the dust–air partition coefficient; instead, it reflects chemical distribution over a given time frame which may be significantly shorter than the time required to achieve equilibrium. The extent to which the dust–air concentration ratios deviate from the dust–air partition coefficients is impacted by various factors including the overall residence time of dust indoors and chemical loss processes.^41^

### Concentrations in Blood and Urine

Figure 2 demonstrates that using RAIDAR-ICE to integrate available environmental measurements from air and dust with dietary intake and dermal application data to calculate aggregate exposures provides blood and urine concentrations that are in good agreement with independent HBM data for a rather diverse range of SVOCs. Both measured and modeled HBM values span approximately 6 orders of magnitude for these chemicals. Overall, the calculated blood and urine concentrations are within 2 orders of magnitude of previously published HBM values for all case study SVOCs and 22/30 (73%) are within 1 order of magnitude. Some notable observations from the comparisons are discussed.

**Figure 2:**
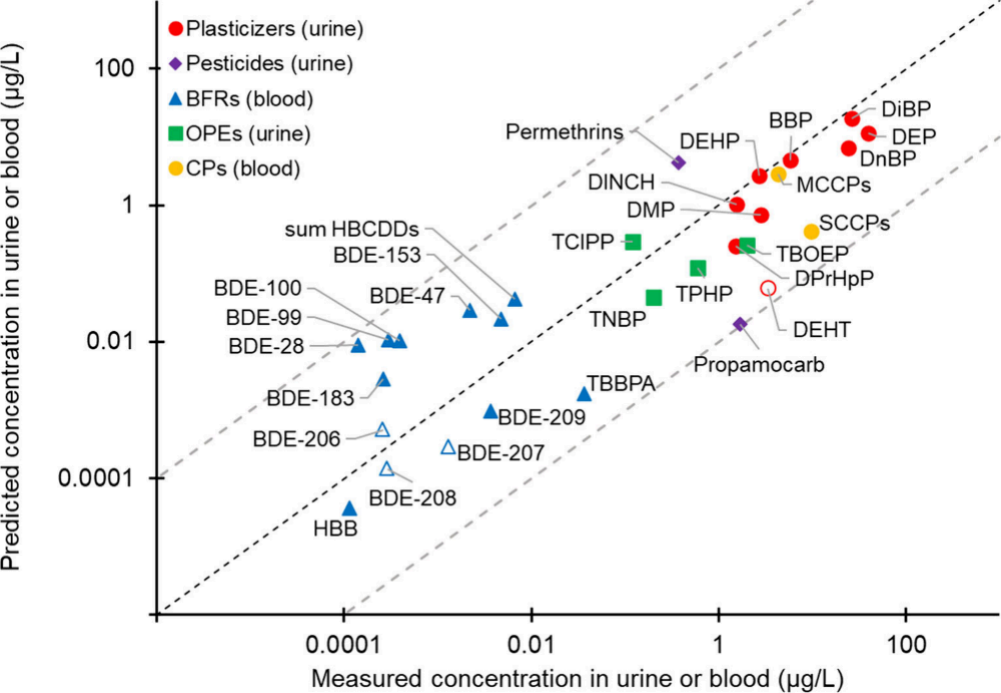
Modeled concentrations of investigated chemicals (see Table S1 for abbreviation definitions) in blood (RAIDAR-ICE toxicokinetic module) and concentrations of metabolites in urine (products of the total daily intake rates calculated by RAIDAR-ICE and *f*_UE_ from the literature, Table S7) based on median measured concentrations in dust and median/mean dietary intake rates and measured median/mean concentrations in blood or urine from the literature (Table S5). Dietary intake was not included in the calculation of DEHT metabolites in urine or BDEs-206, -207, and -208 concentrations in blood (empty markers). Black dashed lines represent agreement between measured and modeled values, and gray dashed lines show values in agreement within ± 2 orders of magnitude.

The calculations reproduce measured MCCP concentrations in human blood but underestimate the blood concentration of SCCP. The discrepancy between calculated and observed SCCP concentrations may result from uncertainty or variability in HBM data, given the analytical challenges caused by the complex composition of commercial CP mixtures containing thousands of congeners.^42,43^ Furthermore, calculated MCCP and SCCP blood concentrations used input parameters reflecting the central tendency of the physicochemical properties for commercial mixtures and reactivity properties selected for the most abundant homologues which were necessary simplifying assumptions that introduce some uncertainty in the calculations.

The calculated urinary concentration of propamocarb is about 2 orders of magnitude lower than the HBM data; however, this comparison is based on very limited and thus uncertain measured HBM data for propamocarb. Propamocarb has been detected in urine in several European countries^44^ but is seldom quantified. The literature value used here was from a pilot study in Sweden that examined the role of organic food diets in mitigating exposure to pesticides.^45^ Of the four urine samples taken during the period of conventional food intake, only one had a quantifiable amount of propamocarb. The authors calculated an estimated daily dietary intake of 0.01 (max 0.2) μg/kg bw/day, which is lower than the EFSA value of 0.39 μg/kg bw/day used in the model calculations (Table S2). Due to the small sample sizes and low detection frequencies, both measurements and modeled data for propamocarb are highly uncertain.

The modeled blood concentration of TBBPA is approximately 20× lower than available HBM data. In a 2011 EFSA review, 652 food samples from multiple European countries were analyzed for TBBPA and all of them were below LOQs (LOQ ≤ 1 ng/g wet weight).^46^ EFSA decided a “worst case” estimate of adult dietary intake of 2.6 ng/kg bw/d, assuming a diet high in fish (208 g/d for an 80 kg adult) with a concentration equal to the maximum LOQ. This is much higher than a previous estimate of 0.04 ng/kg bw/d^47^ from data in The Netherlands and an order of magnitude higher than what was calculated from a Chinese total diet study.^48^ There is therefore significant uncertainty in the dietary intake rates of TBBPA. TBBPA was detected in 36% of serum samples in a Norwegian cohort.^49^ Interestingly, the median concentration reported in the Norwegian study was below the LOQ (0.28 ng/g of lipid), but the geometric mean concentration was much higher (9.4 ng/g of lipid). Support for both values can be found in reported HBM data for TBBPA,^47,50,51^ and therefore it is difficult to determine which value is most representative of the general population. Despite the worst-case dietary intake assumption used in the present study, the modeled concentration in whole blood (0.0017 μg/L) is lower than the reported LOQ in the Norwegian cohort.^49^ This suggests that our understanding of TBBPA exposure and uptake is incomplete. Future investigations should examine the possibility of an additional exposure pathway, for example by dietary contamination from food-contact materials.^52^

Despite the lack of dietary intake data for BDE-206, BDE-207, and BDE-208, calculated blood concentrations for these chemicals were within a factor of 7 or less of measured data. In a study of BDEs in common foods in the U.S., BDEs-206 and -207 were often found at ∼10% the concentration of BDE-209.^53^ No dietary intake of DEHT data was available, and the almost 100-fold underestimation of DEHT metabolites in urine compared to measured values suggests that this pathway could be critical. A previous study found processed food consumption was associated with DEHT exposure.^54^ Figure S3 compares measured to modeled biological concentrations omitting dietary intake for all chemicals and emphasizes the importance of including the dietary pathway in reconciling observed concentration data with calculated outcomes.

Figure 2 does not include comparisons with 1,4-dibromo-2-(2,4-dibromophenoxy)­benzene (BDE-49), DBDPE, and tris­(2-chloroethyl) phosphate (TCEP) because median measured blood concentrations were < LOD (Table S5). Calculated BDE-49 concentrations were almost 4 orders of magnitude lower than the LOD, which is not necessarily an underestimate of empirical concentration estimates as it was detected in less than 2% (3 out of 154) of blood samples in the UK.^55^ DBDPE was detected in 1 out of 55 samples in Sweden^56^ and was not detected in serum in a previous Norwegian study where the LOD was 20 pg/g serum or 7.8 × 10^–5^ μg/L.^57^ The calculated DBDPE blood concentration was 0.0053 μg/L, close to the LOD in the Swedish study. The calculated intake rate of TCEP yields an estimated urinary concentration of its metabolite bis­(2-chloroethyl) phosphate (BCEP) of 0.042 μg/L. This value is < LOD reported in the urinalysis of a German cohort.^58^ This demonstrates how modeling can provide guidance for analytical planning, potentially avoiding unnecessary expenditure of resources on quantifying concentrations that are likely to be below the LODs of the methods used. HBM data that are above detection limits (and preferably for the parent chemical to which humans are exposed) that are colocated with environmental monitoring samples and parametrizing RAIDAR-ICE to the specific exposure conditions could further address uncertainty in human aggregate exposure assessments in the future.

### Relative Contribution of Different Exposure Routes

In addition to reproducing observed chemical concentrations in blood and urine, the holistic modeling framework provides insights into the relative importance of different exposure pathways, which are not readily obtainable through direct monitoring and biomonitoring efforts alone. Figure 3 shows the calculated relative contributions of dietary intake, indoor air inhalation intake, dermal uptake, dust ingestion, and nondust MMI (mouthing mediated ingestion of chemical residues that partition onto indoor surfaces from indoor air but are not bound to dust) to overall exposure. Results were identical for all three stereoisomers of HBCDD, so their sum is presented. Similarly, BDE-208 is representative of BDEs 206, 207, and 208 without dietary ingestion. The aggregate exposure calculations for BDE-208 did not include dietary intake data and dust ingestion was identified as the most important route. Among the other chemicals, dust ingestion accounted for less than 10% of the total exposure with the sole exception being DBDPE (18%). Overall, Figure 3 shows that far-field dietary intake makes an important, if not dominant, contribution to the aggregate human exposure to the investigated chemicals, even though most of these chemicals are predominantly used indoors. While this may seem counterintuitive, previous studies support this finding.^20,29,59^ This is because the majority of the SVOCs have log K_OA_ > 8 and log K_OW_ values between 6 and 9, falling within a chemical partitioning region that leads to significant bioaccumulation in food items. Previous model hypotheses^15^ indicated that dietary ingestion from far-field sources contributes >50% of the aggregate exposure to chemicals within this partitioning region, even if the chemicals are assumed to be released exclusively to the indoor environment. Outside of this partitioning region (e.g., OPEs and phthalates with lower K_OW_), dermal absorption, and other near-field exposure routes may have more importance.

**Figure 3:**
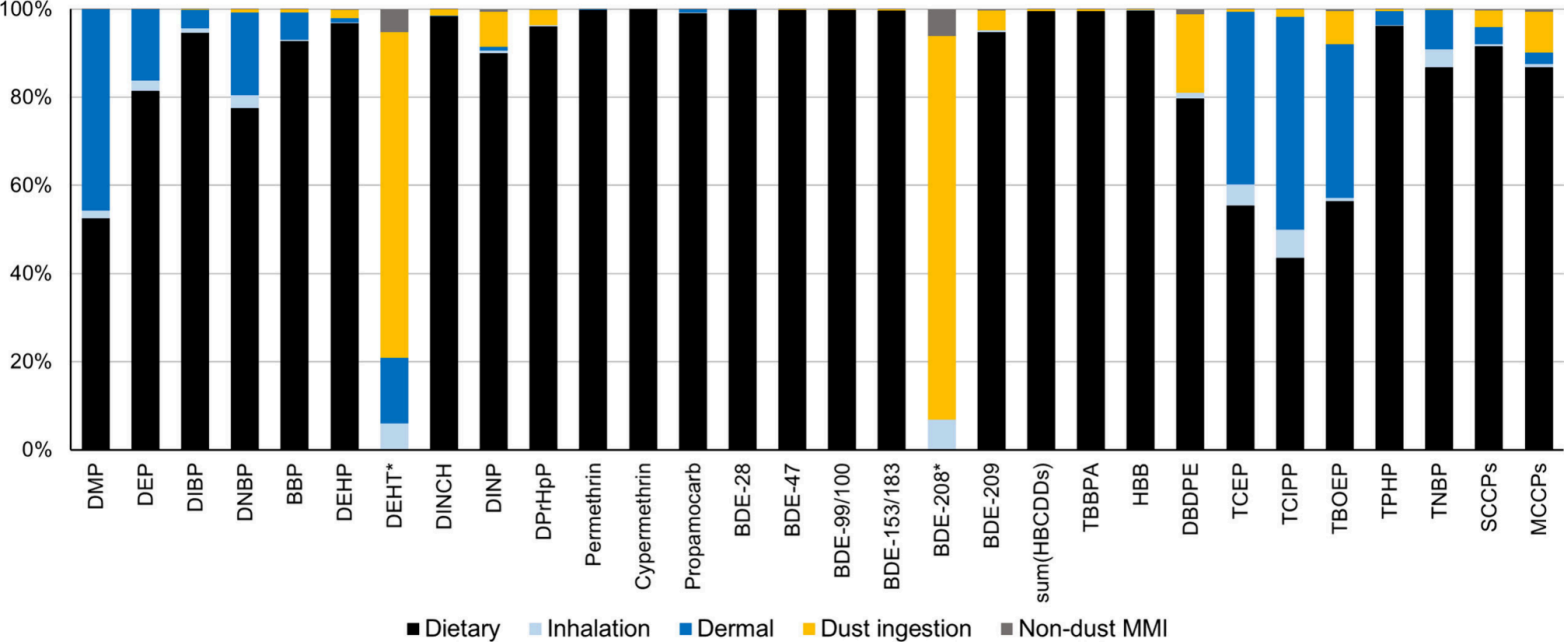
Calculated relative contribution of the major exposure routes: dietary ingestion, inhalation, dermal exposure, mouthing-mediated dust ingestion, and nondust MMI. Direct dermal exposure via PCPs was only considered for DEP. *Dietary intake was not included for DEHT and BDE-208.

For those chemicals for which dietary data were included, far-field dietary exposure was the dominant route, except for tris­(chloro-2-propyl) phosphate (TCIPP) for which dermal uptake was comparable. The importance of dermal exposure for OPEs is supported by a recent analysis of the relative importance of exposure pathways.^20^ In that review, it was determined that dermal uptake of TCEP and TCIPP accounted for 10.5% and 36%, respectively, of the total daily intake for adults, and those quantities are higher for toddlers (93% and 91%, respectively). The previously estimated dietary intake rate of TCIPP was comparable to the one used here, whereas that of TCEP reported here was lower by a factor of 5.4.^60^ It is noted that much higher TCEP concentrations in foodstuffs were measured in several Chinese regions^61,62^ that are not included in the present work which focused on exposures to humans in Europe. For tris­(2-butoxyethyl) phosphate (TBOEP), the model calculates that dietary intake (56%) is the most important exposure route, followed by dermal (35%) and dust ingestion (7.4%) routes. The dominant contribution by dietary intake has also been reported by Gbadamosi et al.,^20^ although their findings suggest inhalation of indoor air plays a more important role (37%) than dermal absorption (11%). In addition, both our work and Gbadamosi et al. agree that total tri-*n*-butyl phosphate (TNBP) intake is dominated by dietary intake but disagree over the importance of dermal exposure versus dietary exposure for triphenyl phosphate (TPHP). Our model calculates that 96% of total TPHP intake is from dietary ingestion, whereas Gbadamosi et al. attribute 38% to dermal routes.

Calculated exposure to DEP arises from both indirect and direct dermal exposure (16%), inhalation (2.3%), and dietary intake (82%). Interestingly, RAIDAR-ICE calculates a total dermal uptake rate of 0.024 μg/kg bw/day without any direct applications of PCPs and 0.061 μg/kg/day including direct applications, meaning even without direct dermal application indirect dermal exposure via the air–skin barrier and skin contact with contaminated surfaces and dust accounts for a significant fraction (6.9%) of overall exposure. Nonetheless, the calculated total exposure, including inhalation, dietary and mouthing-mediated intake and dermal uptake from all sources (0.38 μg/kg/day) is within a factor of 3–4 of other estimates (1.4 ^29^ and 1 μg/kg/day^59^). Wormuth et al.^29^ estimate that dermal uptake from PCP use and mouthing ingestion of PCPs account for more than 80% of total exposure, while Giovanoulis et al.^59^ suggest dietary intake contributes to a little over 60% of the total. The case of DEP highlights the importance of both reliable dermal uptake models and evaluating multiple routes of exposure simultaneously.

### Uncertainty and Variability

Measured exposure data required for exposure and risk assessment are incomplete, and for most chemicals requiring evaluation, measured exposure data are nonexistent. This case study demonstrates the value of combining available monitoring data with mass balance models to address uncertainty and develop holistic aggregate exposure estimates. The results from this case study build confidence in applying models such as RAIDAR-ICE to address data gaps in exposure estimates for chemicals with limited monitoring and human exposure data.

One source of uncertainty is that our work focuses on chemical fate in an average indoor environment and exposure for an average adult, whereas monitoring and biomonitoring data can exhibit inherent and substantial spatial and temporal heterogeneity and interindividual variability and may even lack representativeness for the entire region. For example, the median concentration of DBDPE measured in Irish house dust for the SHINE project was 6,895 ng/g, 100 times higher than the 58 ng/g measured in houses in Belgium. The mechanistic nature of the RAIDAR-ICE model facilitates the analysis of uncertainty in available measured data and many processes relating to human exposures including MMI. A dust ingestion rate of 6.2 mg/day for adults was used for the primary calculations shown in this study, comparable to previously modeled rates,^63,64^ but lower than the value of 30 mg/day (in the 2011 edition) or 20 mg/day (in the 2017 update) in the U.S. EPA’s Exposure Factors Handbook.^65^ Higher dust ingestion rates in the model can be obtained by changing contact frequencies with surfaces and the overall “dustiness” of the environment (resulting from different dust production and cleanup rates). For example, increasing human contact frequency with carpet and vinyl flooring to 24x/day and reducing vacuuming frequency by half increases the dust ingestion rate to 22 mg/day. To explore the uncertainty in the role of dust ingestion on aggregate exposure, we considered some additional analyses. A “high dust” scenario using the third quartile reported concentrations in dust from the highest contaminated locations (Table S8) was used to estimate emission rates and a dust ingestion rate of 22 mg/day was used to calculate intake rates and internal doses. Figures S4 and S5 show the results from the “high dust” scenario calculations. For some chemicals (e.g., BDE-209, TCIPP and TBOEP) assuming a higher concentration in dust and a higher dust ingestion rate results in calculated blood concentrations being greater than the HBM data. Agreement between modeled and measured data was improved for DEHT, TNBP, and TPHP, but only slightly for HBB and TBBPA (Figure S4). In this “high dust” scenario, the calculated DEHT intake was sufficient to account for measured metabolite concentrations in urine, even without dietary intake. For most chemicals in this “high dust” scenario, near-field intake rates are comparable or in some cases higher than dietary intake rates (Figure S5). Higher concentrations in dust corresponded to higher modeled concentrations in indoor air, and the relative importance of inhalation and dermal exposure increased accordingly. Under the “high dust” assumptions, dietary exposure to the OPEs becomes a less significant exposure route. These calculations did not include increased (e.g., third quartile) dietary intake rates and so some caution should be exercised in the comparison of the relative importance of near- and far-field exposure pathways for these chemicals. It is widely accepted that dust ingestion rates for young children and toddlers are even higher than those of adults; therefore, their potential risk of exposure from near-field sources can also be higher.^20,66^ Future modeling efforts should include this age category and its patterns of behavior.

While chemical exposures from dust remain uncertain, the sophisticated mechanistic nature of the RAIDAR-ICE model provides opportunities for addressing these uncertainties by considering different assumptions for MMI within the broader context of aggregate exposure estimation. Moreover, the general applications of the methods and the results shown here emphasize the importance of including all exposure pathways simultaneously when assessing exposure and the capacity to consider different assumptions (e.g., dust ingestion) and data sets (e.g., an Irish house or a Belgian house) for different assessment objectives.

The uncertainties in indoor emission rates and dietary intake rates may be greater than the uncertainty in the dust ingestion rate, and for many chemicals, these uncertainties will more significantly affect the relative importance of different exposure pathways. Integrating the measured data (air, dust, blood, and urine concentrations) with the RAIDAR-ICE model provides an excellent opportunity to assess the relative contribution of different exposure routes (dietary intake, inhalation, dermal exposure, dust ingestion, and nondust MMI) and the impacts of uncertainties and variabilities on the modeled results. Nevertheless, it should be mentioned that the relative importance of exposure pathways may also vary over time due to changes in emissions from multiple lifecycle stages to environmental media as well as evolving environmental conditions. In such cases, time-variant dynamic models such as the PROTEX model may be helpful.^14^

There are uncertainties with model configuration and their required input parameters (e.g., emission rates, physicochemical properties). For example, RAIDAR-ICE characterizes a generic, standardized indoor environment that represents the central tendency of various residential indoor settings but ignores variabilities in indoor characteristics and contents. While this limits the exploration of impacts of indoor characteristics and contents on indoor chemical fate and exposure, we believe that this limitation does not strongly influence the model’s prediction capacity, given earlier empirical evidence that indoor characteristics and contents were not significantly correlated to indoor FR concentrations.^26^ As another example, the model assumes indoor compartments only “receive” chemical emissions and ignores the emissions of chemicals already embedded in indoor compartments (e.g., OPEs from polyurethane foam^37^). This may lead to an underestimation of chemical concentrations and human exposure. For example, earlier observations showed that vinyl flooring was an important product significantly correlated with higher plasticizer concentrations in dust.^24^

Ideally, future monitoring and HBM programs can include spatially and temporally consistent measurements for a range of chemical classes and use categories, and such data can then be used to test models like RAIDAR-ICE and others parametrized specifically to those sampled conditions. Currently such data are not available, and model applications and evaluations like those conducted here can only provide preliminary indications of model performance and guidance for future studies to ultimately obtain a more comprehensive understanding of aggregate exposures to humans.

## Supplementary Material



## Data Availability

The RAIDAR-ICE model can be accessed as part of the Exposure And Safety Estimation (EAS-E) Suite platform: www.eas-e-suite.com.

## Notes

Additional details on model and chemical parameters, dietary intake and HBM data from the literature, and additional results (PDF)
