# Supplementary material for: Integrating Monitoring and Biomonitoring Data with Mechanistic Models to Better Estimate and Characterize Aggregate Human Exposures to Semivolatile Organic Chemicals
Source: Environ Sci Technol. 2025 Dec 2;59(49):26362–72. doi: 10.1021/acs.est.5c08964 (PMC12713766; doi:10.1021/acs.est.5c08964)
Supplement: Supplementary file 1 [file es5c08964_si_001.pdf]

Supporting Information: Integrating monitoring and biomonitoring data with mechanistic models to better estimate and characterize aggregate human exposures to semi-volatile organic chemicals (SVOCs)

Lauren Hughes [a], Jirka Cops [b], Lieve Geerts [b], Katleen De Brouwere [b], Alessandro Sangion [a], Li Li [c], Jon A. Arnot [a,d,e] *

[a] ARC Arnot Research and Consulting Inc., 393 Ashdale Ave Toronto, M4L 2Z3, Canada

[b] Flemish Institute for Technological Research (VITO), Boeretang 200, 2400 Mol, Belgium

[c] School of Public Health, University of Nevada Reno, Reno, Nevada, 89557-274, USA

[d] Department of Pharmacology and Toxicology, University of Toronto, Medical Sciences Building, 1 King's College Cir Toronto, ON, Canada, M5S 3K3

[e] Department of Physical and Environmental Sciences, University of Toronto Scarborough, 1065 Military Trail Toronto, ON, Canada M1C 1A4

* Corresponding author: jon@arnotresearch.com

# of pages: 26

# of tables: 9

# of figures: 5

Table of Contents

RAIDAR-ICE Model Version notes 3

Case Study Chemical Information 4

Calculations for empirical dermal exposure estimates 10

Urinary excretion fraction factors (F_{UE}) 14

Emission rate estimates 16

Supplementary Figures 18

References 23

RAIDAR-ICE Model Version notes

The RAIDAR-ICE model [1] Version 1.8 was used in this work. The RAIDAR-ICE model can be accessed as part of the Exposure And Safety Estimation (EAS-E) Suite platform: www.eas-e-suite.com. A summary of the updates to the RAIDAR-ICE model since v1.10 follows.

Skin cells as a source of particles in the indoor environment: Skin cells have been observed to make up ~75% of dust found in homes [2]. A typical adult human can shed 700-2000 mg skin cells per day [2]. This is significantly higher than the RAIDAR-ICE v1.10 particle emission default. Skin cell loss was already included as a mechanism of chemical loss from the RAIDAR-ICE human, but it was assumed that chemical lost in this way was lost permanently (from the entire indoor environment-human occupant system). In Version 1.8, skin cells lost via natural desquamation are now emitted as particles to the air compartment and included in the dust mass balance (particle sizes 10-150 µm, corresponding to typical "squame" size of 40x30x2 μm^2. This has increased the total particle emission to the room from 288 mg/day to 1460 mg/day (not including particles tracked into the carpet and floor). Particle concentrations on indoor surfaces in version 1.8 are in good agreement with those previously reported [3,4]. The default total suspended particle concentration in RAIDAR-ICE v.1.8 is 29 $\mu g/m^3$, in good agreement with previously measured values[5].

Mouthing-mediated ingestion mechanics: Chemical transfer from room compartments to hands, and from hands to mouth, have been separated into dust-bound and non-dust fractions [3]. Particle (dust) transfer to hands and mouth is now included in the particle mass balance. Previously, dust transfer to the human was assumed to have a negligible effect on the overall mass balance, and dust ingestion rates were inferred from particle concentrations in different media, contact frequencies, and chemical transfer efficiencies. Dust transfer efficiencies and chemical transfer efficiencies are now separate inputs. The previous RAIDAR-ICE model version's default dust ingestion rate (0.026 mg/d) was much lower than calculated values [4] and EPA-recommended assumptions[6] because dust transfer efficiencies were assumed to have the same value as chemical transfer efficiencies, when in fact they should be larger[3], and also because particle emission to the room was underestimated.

Particle dynamics in the indoor environment: Particle size categories and dynamics, including rates of emission, deposition, resuspension, and cleaning have been revisited. New values are based on those documented in Li et al.[3]

PBK model: The RAIDAR-ICE model has been integrated into the EAS-E Suite framework and now uses the same PBK module as the other EAS-E Suite models.

Case Study Chemical Information

Table S1: Chemicals measured in the SHINE project and used in the case study.

Name	Abbreviation	CASRN	SMILES
**Plasticizers**			
Dimethyl phthalate	DMP	131-11-3	COC(=O)C1=CC=CC=C1C(=O)OC
Diethyl phthalate	DEP	84-66-2	CCOC(=O)C1=CC=CC=C1C(=O)OCC
Diisobutyl phthalate	DiBP	84-69-5	CC(C)COC(=O)C1=CC=CC=C1C(=O)OCC(C)C
Dibutyl phthalate	DnBP	84-74-2	CCCCOC(=O)C1=CC=CC=C1C(=O)OCCCC
Butyl benzyl phthalate	BBP	85-68-7	CCCCOC(=O)C1=CC=CC=C1C(=O)OCC2=CC=CC=C2
Di(2-ethylhexyl) phthalate	DEHP	117-81-7	CCCCC(CC)COC(=O)C1=CC=CC=C1C(=O)OCC(CC)CCCC
Bis-(2-ethylhexyl) Terephthalate	DEHT	6422-86-2	CCCCC(CC)COC(=O)C1=CC=C(C=C1)C(=O)OCC(CC)CCCC
Di-isononyl Ester	DINCH	166412-78-8	CC(C)CCCCCCOC(=O)C1CCCCC1C(=O)OCCCCCCC(C)C
Di-isononyl Phthalate	DINP	28553-12-0	CC(C)CCCCCCOC(=O)C1=CC=CC=C1C(=O)OCCCCCCC(C)C
Di-(2-propyl heptyl) Phthalate	DPrHpP	53306-54-0	CCCCCC(CCC)COC(=O)C1=CC=CC=C1C(=O)OCC(CCC)CCCCC
**Pesticides**			
Permethrin	Permethrin	52645-53-1	ClC(=CC1C(C1(C)C)C(=O)OCc1cccc(c1)Oc1ccccc1)Cl
Cypermethrin	Cypermethrin	52315-07-8	N#CC(c1cccc(c1)Oc1ccccc1)OC(=O)C1C(C1(C)C)C=C(Cl)Cl
Propamocarb	Propamocarb	24579-73-5	CCCOC(=O)NCCCN(C)C
**BFRs**			
2,4-dibromo-1-(4-bromophenoxy)benzene	BDE-28	41318-75-6	Brc1ccc(cc1)Oc1ccc(cc1Br)Br
2,4-dibromo-1-(2,4-dibromophenoxy)benzene	BDE-47	5436-43-1	Brc1ccc(c(c1)Br)Oc1ccc(cc1Br)Br
1,4-dibromo-2-(2,4-dibromophenoxy)benzene	BDE-49	243982-82-3	Brc1ccc(c(c1)Br)Oc1cc(Br)ccc1Br
1,2,4-tribromo-5-(2,4-dibromophenoxy)benzene	BDE-99	60348-60-9	Brc1ccc(c(c1)Br)Oc1cc(Br)c(cc1Br)Br
1,3,5-tribromo-2-(2,4-dibromophenoxy)benzene	BDE-100	189084-64-8	Brc1ccc(c(c1)Br)Oc1c(Br)cc(cc1Br)Br
1,2,4-tribromo-5-(2,4,5-tribromophenoxy)benzene	BDE-153	68631-49-2	Brc1cc(Br)c(cc1Oc1cc(Br)c(cc1Br)Br)Br
1,2,3,5-tetrabromo-4-(2,4,5-tribromophenoxy)benzene	BDE-183	207122-16-5	Brc1cc(Br)c(cc1Oc1c(Br)cc(c(c1Br)Br)Br)Br

Name	Abbreviation	CASRN	SMILES
1,2,3,4,5-pentabromo-6-(2,3,4,5-tetrabromophenoxy)benzene	BDE-206	63387-28-0	Brc1cc(Oc2c(Br)c(Br)c(c(c2Br)Br)Br)c(c(c1Br)Br)Br
1,2,3,4,5-pentabromo-6-(2,3,4,6-tetrabromophenoxy)benzene	BDE-207	437701-79-6	Brc1c(Br)cc(c(c1Br)Oc1c(Br)c(Br)c(c(c1Br)Br)Br)Br
1,2,3,4,5-pentabromo-6-(2,3,5,6-tetrabromophenoxy)benzene	BDE-208	437701-78-5	Brc1c(Oc2c(Br)c(Br)cc(c2Br)Br)c(Br)c(c(c1Br)Br)Br
1,2,3,4,5-pentabromo-6-(2,3,4,5,6-pentabromophenoxy)benzene	BDE-209	1163-19-5	Brc1c(Oc2c(Br)c(Br)c(c(c2Br)Br)Br)c(Br)c(c(c1Br)Br)Br
α-hexabromocyclododecane	α_HBCDD	134237-50-6	BrC1CCC(Br)C(Br)CCC(C(CCC1Br)Br)Br
β-hexabromocyclododecane	β_HBCDD	134237-51-7	BrC1CCC(Br)C(Br)CCC(C(CCC1Br)Br)Br
γ-hexabromocyclododecane	γ_HBCDD	134237-52-8	BrC1CCC(Br)C(Br)CCC(C(CCC1Br)Br)Br
3,3′,5,5′-tetrabromobisphenol A	TBBPA	79-94-7	CC(c1cc(Br)c(c(c1)Br)O)(c1cc(Br)c(c(c1)Br)O)C
hexabromobenzene	HBB	87-82-1	Brc1c(Br)c(Br)c(c(c1Br)Br)Br
1,1'-(ethane-1,2-diyl)bis[pentabromobenzene]	DBDPE	84852-53-9	Brc1c(CCc2c(Br)c(Br)c(c(c2Br)Br)Br)c(Br)c(c(c1Br)Br)Br
**OPEs**			
Tris(2-chloroethyl) phosphate	TCEP	115-96-8	ClCCOP(=O)(OCCCl)OCCCl
Tris(chloro-2-propyl) phosphate	TCIPP	13674-84-5	ClCC(OP(=O)(OC(CCl)C)OC(CCl)C)C
Tris(2-butoxyethyl) phosphate	TBOEP	78-51-3	CCCCOCCOP(=O)(OCCOCCCC)OCCOCCCC
Triphenyl phosphate	TPHP	115-86-6	O=P(Oc1ccccc1)(Oc1ccccc1)Oc1ccccc1
Tri-n-butyl phosphate	TNBP	126-73-8	CCCCOP(=O)(OCCCC)OCCCC
**CPs**			
Short chain chlorinated paraffins (C10-13)	SCCP	85535-84-8	CCC(C(CC(CC(C(C(CC)Cl)Cl)Cl)Cl)Cl)Cl
Medium-chain chlorinated paraffins (C14-17)	MCCP	85535-85-9	CCCC(C(CC(CC(C(C(C(CC)Cl)Cl)Cl)Cl)Cl)Cl)Cl

Table S2: Concentrations in air and dust measured in the SHINE project (medians, ranges, and detection frequencies (DF)), and the RAIDAR-ICE-estimated emission rates to air (E_{Air}). Emission rates were calculated from the median concentrations in dust for all chemicals except for DMP, for which the median concentration in air was used.

	Measured concentration in dust (ng/g)			Measured concentration in air (ng/m^3)			E_{Air} (ng/m^{2}h)
	median	range	DF (%)	median	range	DF (%)	
**Plasticizers**							
DMP	20	< 200 – 5600	11	2.4	< 3 – 23	50	3.17E+00
DEP	390	< 200 – 6600	83	47	13 – 117	100	7.79E+01
DiBP	4800	< 200 – 1.5 × 10^5	98	67	35 – 265	100	5.66E+01
DnBP	6800	2000 – 2.3 × 10^5	100	86	21 – 367	100	5.58E+01
BBP	4700	< 200 – 2.0 × 10^5	98	0.60	< 3 – 5	20	6.09E+00
DEHP	1.1 × 10^5	9000 – 2.0 × 10^6	100	11	7 – 38	100	7.19E+01
DEHT	3.8 × 10^4	5100 – 7.6 × 10^5	100	110	< 10 – 623	90	2.49E+01
DINCH	1.3 × 10^4	< 5000 – 1.1 × 10^6	96	0.50	< 5 - 7	10	8.45E+00
DINP	6.3 × 10^4	< 5000 – 1.9 × 10^6	98	10	< 5 - 8	20	4.09E+01
DPrHpP	4.1 × 10^3	< 1000 – 9.0 × 10^4	94	n.d.	< 5	0	2.66E+00
**Pesticides**							
Permethrin	280	< 3.8 – 7600	53				1.96E-01
Cypermethrin	21	< 3.2 – 530	12				1.38E-02
Propamocarb	87	< 2.1 – 180	28				6.24E+00
**BFRs**							
BDE-28	0.050	< 0.044 – 3.3	36	1.4 × 10^{-4}	< 0.00035 – 0.0039	40	1.77E-04
BDE-47	3.6	< 0.2 – 240	64	5.8 × 10^{-3}	< 0.002 – 0.061	80	3.46E-03
BDE-49	0.20	< 0.09 – 5.3	73	1.0 × 10^{-4}	< 1.4 × 10^{-5} – 0.0012	90	2.33E-04
BDE-99	2.0	< 0.2 – 480	53	1.3 × 10^{-4}	< 0.00053 - 0.0063	25	1.41E-03
BDE-100	0.86	< 0.2 – 83	40	7.2 × 10^{-5}	< 0.00036 – 0.003	20	6.32E-04
BDE-153	1.5	< 0.2 – 130	76	n.d.	-	0	9.96E-04
BDE-183	0.70	< 0.2 – 240	56	n.d.	-	0	4.57E-04
BDE-206	8.2	< 2.8 – 56	87	2.4 × 10^{-5}	< 0.00024 – 0.0018	10	5.33E-03
BDE-207	5.0	2 – 26	100	n.d.	-	0	3.25E-03

	Measured concentration in dust (ng/g)			Measured concentration in air (ng/m^3)			E_{Air} (ng/m^{2}h)
	median	range	DF (%)	median	range	DF (%)	
BDE-208	2.9	1 - 9	100	n.d.	-	0	1.88E-03
BDE-209	240	1 – 7.0 × 10^4	100	0.003	< 0.0012 – 2.0	30	1.56E-01
α-HBCDD	56	< 0.5 – 5.1 × 10^4	98	4.0 × 10^{-4}	< 0.00024 – 1.5	40	3.68E-02
β-HBCDD	16	< 0.25 – 4.8 × 10^4	89	2.7 × 10^{-3}	< 0.00030 – 1.9	50	1.05E-02
γ-HBCDD	27	1 - 4700	100	4.5 × 10^{-4}	< 0.00038 – 1.8	45	1.77E-02
TBBPA	45	< 0.5 - 5600	82	n.d.		0	2.92E-02
HBB	1.0	< 0.2 - 101	70	0.056	< 0.010 – 0.85	85	2.92E-03
DBDPE	77	< 11 – 1.3 × 10^6	68	1.3	< 0.00090 – 3000	55	4.99E-02
**OPEs**							
TCEP	380	< 120 – 8.2 × 10^4	97	0.29	120 – 38.2	90	5.02E+00
TCIPP	3600	< 340 – 1.3 × 10^6	98	38	0.15 – 245	100	2.73E+01
TBOEP	1.4 × 10^4	93[a] – 7.5 × 10^6	98	0.18	< 0.00084 – 0.61	70	9.23E+00
TPHP	800	31[a] – 1.7 × 10^5	98	0.057	< 0.025 – 0.424	95	1.13E+00
TNBP	41	< 2.5 – 6400	47	2.2	0.97 – 7.1	100	2.67E+00
**CPs**							
SCCPs	3200	n.r.[b] – 5.5 × 10^4					2.27E+00
MCCPs	1.8 × 10^4	n.r.[b] – 3.5 × 10^5					1.19E+01

[a] LODs differed between locations and reported LODs in some locations were much higher than minimum measured values in others.

[b] Not reported

Table S3: Dietary intake rates used in RAIDAR-ICE simulations.

Substance	Reported dietary intake (µg/kg/d)	Dietary intake rate for 80 kg adult (µg/d)	Details	Reference
**Plasticizers**				
DMP	1.19E-02	9.52E-01	Geometric mean; Norway	[7]
DEP	3.09E-01	2.47E+01	Geometric mean; Norway	[7]
DiBP	6.38E-01	5.10E+01	Geometric mean; Norway	[7]
DnBP	1.60E-01	1.28E+01	Mean; Belgium	[8]
BBP (BBzP)	1.42E-01	1.14E+01	Geometric mean; Norway	[7]
DEHP	1.26E+00	1.01E+02	Geometric mean; Norway	[7]
DEHT	no data	5.36E+01		
DINCH	2.15E-01	1.72E+01	Geometric mean; Norway	[7]
DINP	1.63E-01	1.30E+01	Geometric mean; Norway	[7]
DPrHpP	2.75E-02	2.20E+00	Geometric mean; Norway	[7]
**Pesticides**				
Permethrin	1.25E-01	1.00E+01	Europe [a]	[9]
Cypermethrin	3.95E-01	3.16E+01	Europe [a]	[9]
Propamocarb	3.90E-01	3.12E+01	Europe [a]	[9]
**BFRs**				
BDE-28	9.50E-05	7.60E-03	Europe [b]	[10]
BDE-47	6.50E-04	5.20E-02	Europe [b]	[10]
BDE-49	no data	0		
BDE-99	2.65E-04	2.12E-02		[10]
BDE-100	2.25E-04	1.80E-02	Europe [b]	[10]
BDE-153	1.50E-04	1.20E-02	Europe [b]	[10]
BDE-183	1.25E-04	1.00E-02	Europe [b]	[10]
BDE-206	no data	0		
BDE-207	no data	0		
BDE-208	no data	0		
BDE-209	1.15E-03	9.20E-02	Europe [b]	[10]
α-HBCDD		2.03E-01	Mean and median; UK	[11]
β-HBCDD		1.05E-01	Mean and median; UK	[11]

Substance	Reported dietary intake (µg/kg/d)	Dietary intake rate for 80 kg adult (µg/d)	Details	Reference
γ-HBCDD		1.12E-01	Mean and median; UK	[11]
TBBPA	2.6E-03	2.1E-01	"Worst case"; Europe	[12]
HBB	1.9E-04	1.5E-02	Average; Latvia	[13]
DBDPE	8.1E-05	6.48E-03	Average; UK	[14]
**OPEs**				
TCEP	6.00E-03	4.80E-01	Mean; Sweden	[15]
TCIPP	1.85E-02	1.48E+00	Mean (medium bound); Belgium	[15]
TBOEP	2.20E-02	1.76E+00	Average; UK	[16]
TPHP	4.66E-02	3.73E+00	Mean (medium bound); Belgium	[15]
TNBP	5.50E-03	4.40E-01	Mean (medium bound); Belgium	[15]
**CPs**				
SCCPs	1.80E-02	1.44E+00	Mean; Sweden	[17]
MCCPs	3.90E-02	3.12E+00	Mean; Sweden	[17]

[a,b] Average of upper and lower bound estimates

Calculations for empirical dermal exposure estimates

Product concentration and application information for DEP were retrieved from Wormuth et al. [18] and the total application rate, E_{skin} (ng/h), was calculated as $E_{skin} = C_{prod} \times q_{prod} \times f_{skin} \times f_{event}/24$ where C_{prod} is the concentration of DEP in the product (ng/g), q_{prod} is the mean volume of product per application (g), f_{skin} is the fraction retained on skin after product use and f_{event} is the mean number of applications of the product per day ($f_{event} < 1$ for a product that is applied less frequently than once daily). **Table S4** summarizes the data used in this case study.

Table S4: Product application rate to skin for selected phthalates [18].

	**Application rate (ng/h)**		
	**DMP**	**DEP**	**DnBP**
Deodorant	10.7	6.28	2.01
Perfumes	15.1	32.9	1.04
Aftershave	14.0	62.9	38.2
Hair styling product	3.65	0.075	0.0305
Shampoo	10.1	2.71	0.152
Skin care	0	55.8	0
**Total**	**53.5**	**160.6**	**41.4**

Table S5: Human biomonitoring data for selected chemical substances.

Substance	Concentration (µg/l)	Details	Type of data	Medium	Geographical region	Reference
**Plasticizers**						
**DMP**	2.8	Metabolite (MMP)	Median	Urine	Germany	[19]
**DEP**	3.94E+01	Metabolite (MEP)	Reference mean	Urine	Belgium	[20]
**DiBP**	2.61E+01	Metabolite (MiBP)	Reference mean	Urine	Belgium	[20]
**DnBP**	2.43E+01	Metabolite (MnBP)	Reference mean	Urine	Belgium	[20]
**BBP (BBzP)**	5.74E+00	Metabolite (MBzP)	Reference mean	Urine	Belgium	[20]
**DEHP**	2.70E+00	Metabolite (MEHP)	Reference mean	Urine	Belgium	[20, 21]
**DEHT**	3.35E+00	Metabolite (5cx MEPTP)	Median	Urine	Germany	[22]
**DINCH**	1.17 OH-MINCH<br>0.37 cx-MINCH	Median 24-h urine, mixed adults	Median	Urine	Germany	[23]
**DPrHpP**	0.8 OH-MiDP<br>0.3 oxo-MiDP<br>0.4 cx-MiDP	Median 24-h urine, mixed adults [a]	Median	Urine	Germany	[19]
**Pesticides**						
**Permethrin/ Cypermethrin**	0.37	Sum of DCCA and 3-PBA	Median	Urine	Sweden	[24]
**Propamocarb**	1.7	1 of 4 samples > LOQ, taken from a single adult male)	Median	Urine	Sweden	[25]
**BFRs**						
**BDE-28**	1.4E-04	0.036 ng/g lipid in serum	Median	Blood	Sweden	[26]
**BDE-47**	2.2E-03	0.56 ng/g lipid in serum	Median	Blood	Sweden	[26]
**BDE-49**	< 2.3E-04	< 0.059 ng/g lipid in serum	Median	Blood	Norway	[27]
**BDE-99**	3.0E-04	0.078 ng/g lipid in serum	Median	Blood	Netherlands	[26]
**BDE-100**	3.9E-04	0.1 ng/g lipid in serum	Median	Blood	Sweden	[26]
**BDE-153**	4.7E-03	1.2 ng/g lipid in serum	Median	Blood	Sweden	[28]

Substance	Concentration (µg/l)	Details	Type of data	Medium	Geographical region	Reference
**BDE-183**	2.6E-04	0.066 ng/g lipid in serum	Median	Blood	UK	[29]
**BDE-206**	2.6E-04	0.066 ng/g lipid in serum	Median	Blood	Sweden	[26]
**BDE-207**	1.3E-03	0.33 ng/g lipid in serum	Median	Blood	Sweden	[26]
**BDE-208**	2.9E-04	0.074 ng/g lipid in serum	Median	Blood	Sweden	[26]
**BDE-209**	3.7E-03	0.95 ng/g lipid in serum	Median	Blood	Sweden	[28]
**Sum HBCDDs**	6.6E-03	1.7 ng/g lipid in serum	Median	Blood	Netherlands	[30]
**TBBPA**	3.6E-02	9.4 ng/g lipid in serum	Geo. mean	Blood	Norway	[27]
**HBB**	1.16E-04	0.03 ng/g lipid in serum	Median	Blood	Norway	[31]
**DBDPE**	< 6.60E-03	< 12 ng/g lipid in serum	Median	Blood	Sweden	[32]
**OPFRs**						
**TCEP**	< 0.1	Metabolite (BCEP)	Median	Urine	Germany	[33]
**TCIPP**	0.12	Metabolite (BCIPP)	Median	Urine	Belgium	[34]
**TBOEP**	2.00E+00	Metabolite (BBOEP)	Median	Urine	Germany	[35]
**TPHP**	0.59	Metabolite (DPHP)	Median	Urine	Belgium	[34]
**TnBP**	2.00E-01	Metabolite (DnBP)	Median	Urine	Germany	[35]
**CPs**						
**SCCPs**	9.69	2500 ng/g lipid in serum	Median	Blood	Norway	[36]
**MCCPs**	4.26	1100 ng/g lipid in serum	Median	Blood	Norway	[36]

[a] The analytical method used could not distinguish between DPrHpP and di-isodecyl phthalate (DiDP) metabolites.

Table S6: RAIDAR-ICE model chemical property input parameters. Recommended values from EAS-E Suite (accessed 2024-03-05) were chosen unless otherwise noted. Reaction rates on surfaces are assumed to be negligible (HL = 10^{12} h).

	Molar mass (g/mol)	$logK_{OW}$	$logK_{OA}$	HL in indoor air (h)	HL_B (h)
**Plasticizers**					
DMP	194.18	1.58	6.82	611	2.19
DEP	222.24	2.45	7.68	203	2.24
DiBP	278.35	4.11	8.94	98.0	4.42
DnBP	278.35	4.61	9.11	97.9	2.62
BBP	312.37	4.82	10.22	91.2	0.253
DEHP	390.57	7.53	12.85	53.7	0.744
DEHT	390.57	9.12	12.39	35.1	3.58
DINCH	424.67	10.36	13.26	36.9 [a]	2.24
DINP	418.62	9.56	13.96	32.9	2.71
DPrHpP	446.66	10.79	14.01	37.8 [a]	3.49
**Pesticides**					
Permethrin	391.30	6.50	11.39	40.4	37.1
Cypermethrin	416.31	6.60	12.47	41.2	26.1
Propamocarb	188.27	1.12	8.20	7.80	2.13
**BFRs**					
BDE-28	406.90	5.94	9.5	545	33900
BDE-47	485.80	6.81	10.53	768	15500
BDE-49	485.80	6.29	10.30	762	532
BDE-99	564.69	7.32	11.31	1400	14100
BDE-100	564.69	7.24	11.13	1070	16600
BDE-153	643.59	7.9	11.96	3320	63100
BDE-183	722.48	8.27	12.78	4630	7760
BDE-206	880.28	8.3	14.44	11600	140000
BDE-207	880.28	8.3	14.44	10200	120000
BDE-208	880.28	8.3	14.44	11600	90700
BDE-209	959.17	8.7	15.27	22900	288
α_HBCDD	641.70	7.17	12.31	154	4070
β_HBCDD	641.70	7.17	12.31	154	1320
γ_HBCDD	641.70	7.17	12.31	154	1320
TBBPA	543.88	7.36	15.49	260	214
HBB	551.49	6.07	9.63	67300	58.6
DBDPE	971.23	12.97	18.84	1110 [a]	138000
**OPEs**					
TCEP	285.49	1.44	8.90	35.0	27.2
TCIPP	327.57	2.59	9.18	17.2	42.5
TBOEP	398.48	3.75	13.03	5.98	1.86
TPHP	326.29	4.59	10.14	71.0	9.23
TNBP	266.32	4.00	8.23	9.77	2.07
**CPs** [b]					
SCCPs	377.01	6.78	11.3	464	7030
MCCPs	439.51	7.27	12.2	344	24100

[a] Calculated from consensus (geometric mean) of k_{OH} values calculated by OPERA (retrieved by Comptox Chemicals Dashboard) and AOPWIN[37], assuming an indoor OH concentration of 2.5E+05 molecules/cm^3

[b] Represented by homologs (C13Cl6 and C14Cl7, respectively) with both (i) the highest abundance in commercial SCCP and MCCP mixtures and (ii) the partitioning properties closest to those of the commercial SCCP and MCCP mixtures (according to measurements reported in the EU Risk Assessment Reports)[38, 39].

Urinary excretion fraction factors (F_{UE})

Table S7 lists the urinary excretion fraction factors (F_{UE}) used in this study based on methods and assumptions detailed elsewhere [40, 41]. Briefly, concentrations of metabolites in urine were calculated using the FUE equation:

$$C_{urine} = DI / [(UV_{excr} / F_{UE}) \times (MW_{parent} / MW_{metabolite})]$$

where C_{urine} (µg/l) is the concentration of the metabolite in the urine, DI (µg/kg-bw/day) is the total daily intake of parent chemical, UV_{excr} (L/kg-bw/day) the excreted urine volume (0.02 L/kg-bw/day for adults) [42], MW_{parent} and $MW_{metabolite}$ (g/mol) are the molecular weights of the parent compound and the metabolite respectively, and F_{UE} is the molar fraction of urine-excreted metabolite with respect to the administered dose of its parent compound. Since permethrin and cypermethrin have three common metabolites (cis-DCCA, trans-DCCA and 3-PBA), the external exposures to permethrin and cypermethrin were summed for comparison with HBM data, as the metabolites could not be attributed to either one or the other.

Table S7: Urinary excretion fraction factors (F_{UE}).

Parent compound	Metabolite	F_{UE} factor	Source F_{UE} value*
**Plasticizers**			
DMP	MMP	0.69	[43]
DEP	MEP	0.69	[43]
DiBP	MIBP	0.703	[44]
DnBP	MnPB	0.84	[44]
BBP (=BBzP)	MBzP	0.73	[43]
DEHP	MEHP	0.059	[45]
DEHT	5cx-MEPTP	0.1295	[46]
DINCH	OH-MINCH	0.1073	[47]
	cx-MINCH	0.0203	
DPrHpP	OH-MPHP	0.107	[48]
	cx-MPHP	0.0048	
	oxo-MPHP	0.1352	
**Pesticides**			
Permethrin/Cypermethrin	Cis-DCCA	0.07	[49]
	trans-DCCA	0.15	
	3-PBA	0.094	
**OPFRs**			
TCEP	BCEP	0.10	[50]
TCIPP	BCIPP	0.18	[51]
TBOEP	BBOEP	0.18	[51]
TPHP	DPHP	0.065	[50]
TNBP	DNBP	0.18	[51]

***F_{UE}** factor is mentioned as 'FUE factor' in literature or is otherwise derived from data in the publication. In case of multiple F_{UE} factors for one compound, the mean of reported values has been used.

Table S8: Concentrations in dust for the highest contaminated settings in the SHINE project.

	**Concentrations in dust (ng/g)**			**Country (setting)**
	median	mean	3rd quartile	
DnBP	25000	57000	78000	Sweden (offices)
BBP	46000	74000	78000	Sweden (preschools)
DEHP	197000	751000	1352000	Sweden (offices)
DEHT	164000	151000	247000	Ireland (houses)
DINP	287000	648000	926000	Sweden (offices)
BDE 209	12955	20146	18800	Ireland (houses)
α_HBCDD	681	5764	1230	Ireland (houses)
β_HBCDD	247	5213	823	Ireland (houses)
γ_HBCDD	2300	13477	4680	Ireland (houses)
TBBPA	490	1009	990	Netherlands (offices)
HBB	26	34	40	Ireland (houses)
DBDPE	6895	24110	18780	Ireland (houses)
TCEP	3330	11301	6080	Ireland (houses)
TCIPP	25100	43766	72600	Netherlands (offices)
TBOEP	1870000	2070000	2940000	Netherlands (offices)
TPHP	3495	30462	24300	Ireland (houses)
TNBP	1200	2140	2000	Sweden (preschools)

Emission rate estimates

Table S9. Total indoor emission rate estimates derived from inverse modeling with European data (this study) and American data[3] for 24 case study chemicals, and emissions from specific materials and objects calculated from chamber experiments and emission modeling.

	**Back-calculated from concentrations in dust ($ng\ m^{-2}\ h^{-1}$)**		**Previous experiments and modeling ($ng\ m^{-2}\ h^{-1}$, unless otherwise noted)**	
Chemical	This study	Li et al., 2021		**Ref.**
DEP	78		N.D. - 790 (synthetic leather)<br>N.D. - 840 (wallpaper)	52
DiBP	57	0.74	N.D. - 1100 (synthetic leather)<br>N.D. - 290 (wallpaper)<br>N.D. - 91 (vinyl flooring)	52
DnBP	56	22	N.D. – 320 (synthetic leather)<br>N.D. -770 (wallpaper)<br>N.D. – 430 (vinyl flooring)	52
BBP	6.1	32	N.D. – 4400 (wallpaper)	52
DEHP	72	24	192 (vinyl flooring, no particles)<br><br>N.D. – 3.2×10^4 (synthetic leather)<br>N.D. – 6900 (wallpaper)<br>N.D. – 3.0×10^4 (vinyl flooring)	53<br><br>52
DEHT	25	22		
DINP	41	14		
BDE-28	1.8×10^{-4}	6.1×10^{-3}	1.9 ng $unit^{-1}\ h^{-1}$ (PCB, mfg. date 2000, at 60°C)	54
BDE-47	3.5×10^{-3}	0.27	14.2 ng $unit^{-1}\ h^{-1}$ (PCB, mfg. date 2000, at 60°C)	54
BDE-49	2.3×10^{-4}	3.7×10^{-3}		
BDE-99	1.4×10^{-3}	0.18	2.6 ng $unit^{-1}\ h^{-1}$ (PCB, mfg. date 2000, at 60°C)	54
BDE-100	6.3×10^{-4}	3.0×10^{-2}	1.3 ng $unit^{-1}\ h^{-1}$ (PCB, mfg. date 2000, at 60°C)	54
BDE-153	1.0×10^{-4}	1.8×10^{-2}	0.04 ng $unit^{-1}\ h^{-1}$ (PCB, mfg. date 2000, at 60°C)	54
BDE-183	4.6×10^{-4}	1.4×10^{-2}		
BDE-207	3.3×10^{-3}	2.1×10^{-2}		
BDE-208	1.9×10^{-3}	1.0×10^{-2}		
BDE-209	0.16	0.46		
α-HBCDD	3.7×10^{-2}	7.9×10^{-2}		
β-HBCDD	1.1×10^{-2}	6.1×10^{-3}		
γ-HBCDD	1.8×10^{-2}	1.9×10^{-2}		
TCEP	5.0	6.7	695; 118 (wooden floorboard)<br>21.3; 10.0 (wallpaper)<br>142; 1100 (latex paint)	55

			18.9; 261; 148 (carpet)<br>151; 248; 324 (“decorative leather”)	
TCIPP	27	49	20 – 1.4 × 10^5 (PUF)<br><br>361; 371 (wooden flooring)<br>49.0; 79.2 (wallpaper)<br>39.9 (latex paint)<br>51.5; 164 (carpet)<br>216; 105; 16.0 (“decorative leather”)	[54]<br><br>[55]
TBOEP	9.2	34	82.2 (wooden floorboard)<br>32.7; 32.9 (wallpaper)<br>70.2 (“decorative leather”)	[55]
TPHP	1.1	0.43	29.7 (wooden floorboard)<br>2.6 (wallpaper)	[55]
TNBP	2.7		212; 1090 (wooden floorboard)<br>14.1; 24.6 (wallpaper)<br>46.0; 38.8 (latex paint)<br>62.7; 135 (carpet)<br>157; 35.1; 20.9 (“decorative leather”)	[55]

PCB: Printed circuit board; PUF: polyurethane foam

Supplementary Figures

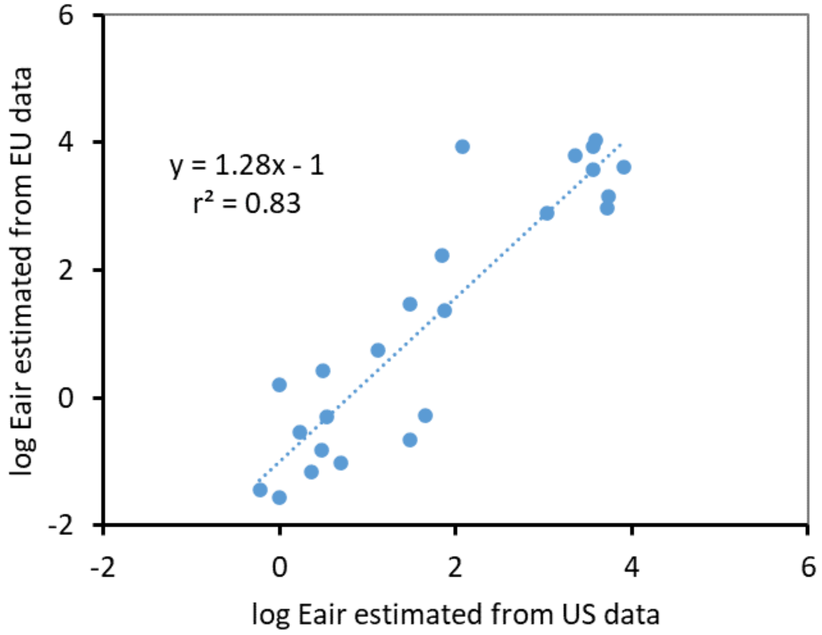

Figure S1: Comparison of inverse modeling estimates for chemical emission rates to air (ng/h) from this study using EU data and from a previous study using data from North America [3] for 24 case study chemicals.

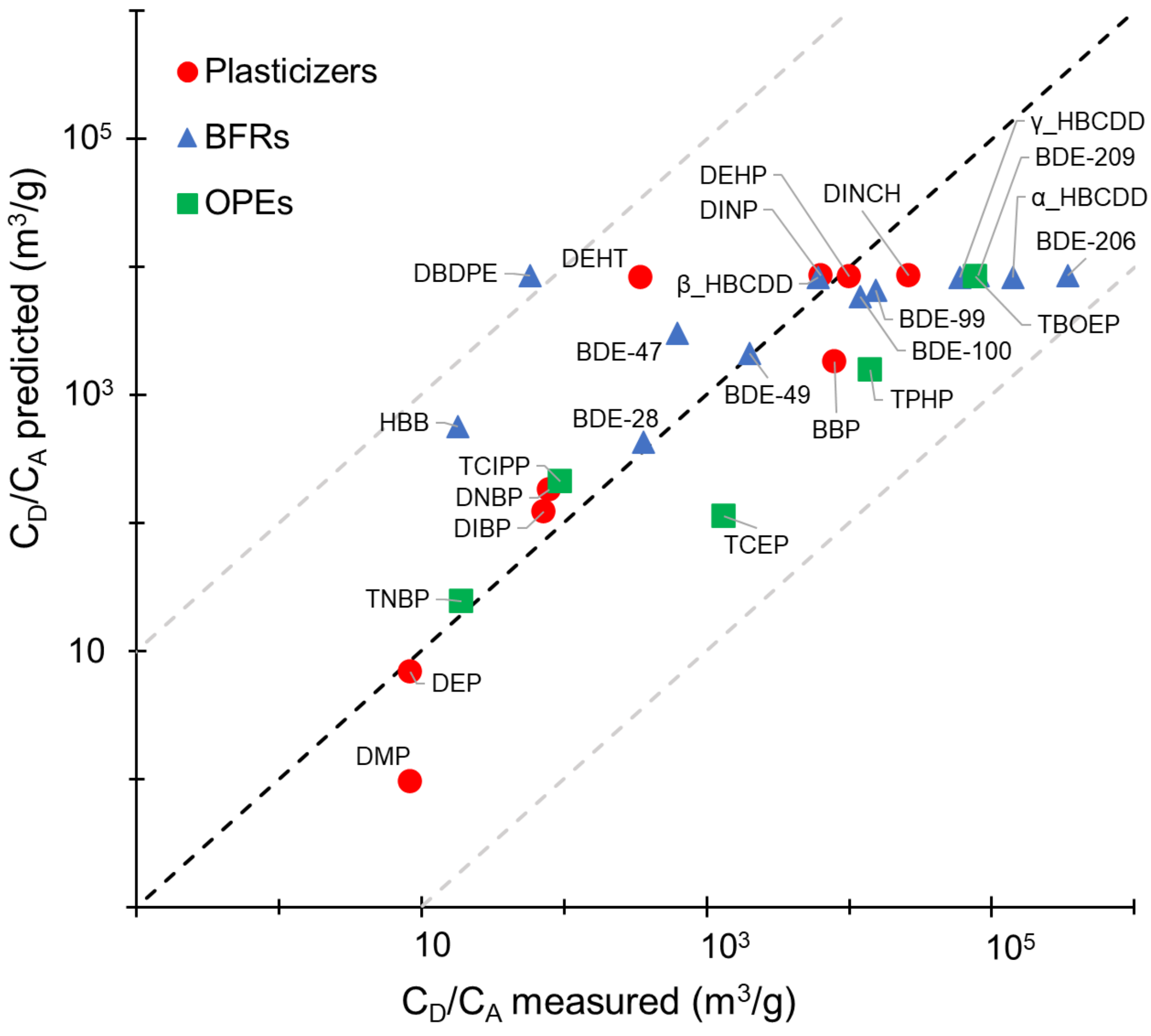

Figure S2: Dust-air concentration ratios (C_D/C_A) for plasticizers, brominated flame retardants (BFRs), and organophosphate esters (OPEs), calculated based on RAIDAR-ICE's default parameterization vs. calculated from median measured concentrations in dust and air collected from the SHINE project. Black dashed lines represent agreement between measured and modeled values, and grey dashed lines show values in agreement within ± two orders of magnitude.

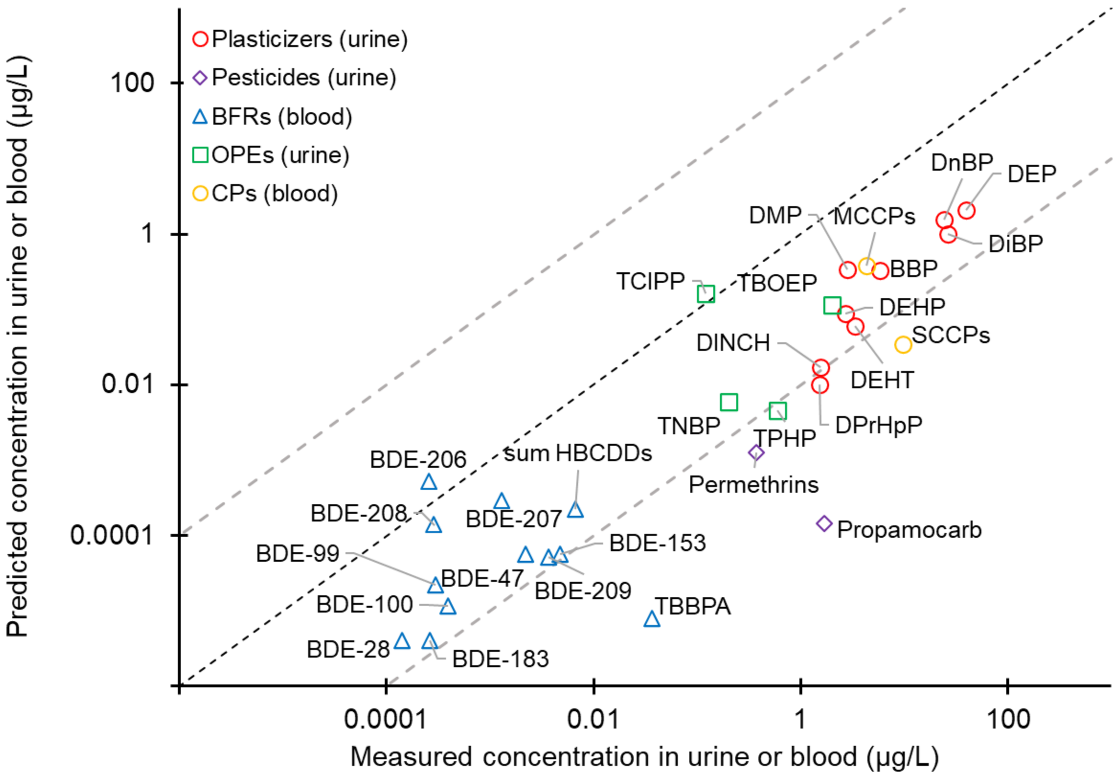

Figure S3: Modeled concentrations of investigated chemicals in blood (RAIDAR-ICE toxicokinetic module) and concentrations of metabolites in urine (products of the total daily intake rates calculated by RAIDAR-ICE and f_{UE} from the literature, **Table S7**) based on median measured concentrations in dust and excluding dietary intake rates, and measured median/mean concentrations in blood or urine from the literature (**Table S5**). Black dashed lines represent agreement between measured and modeled values, and grey dashed lines show values in agreement within ± two orders of magnitude.

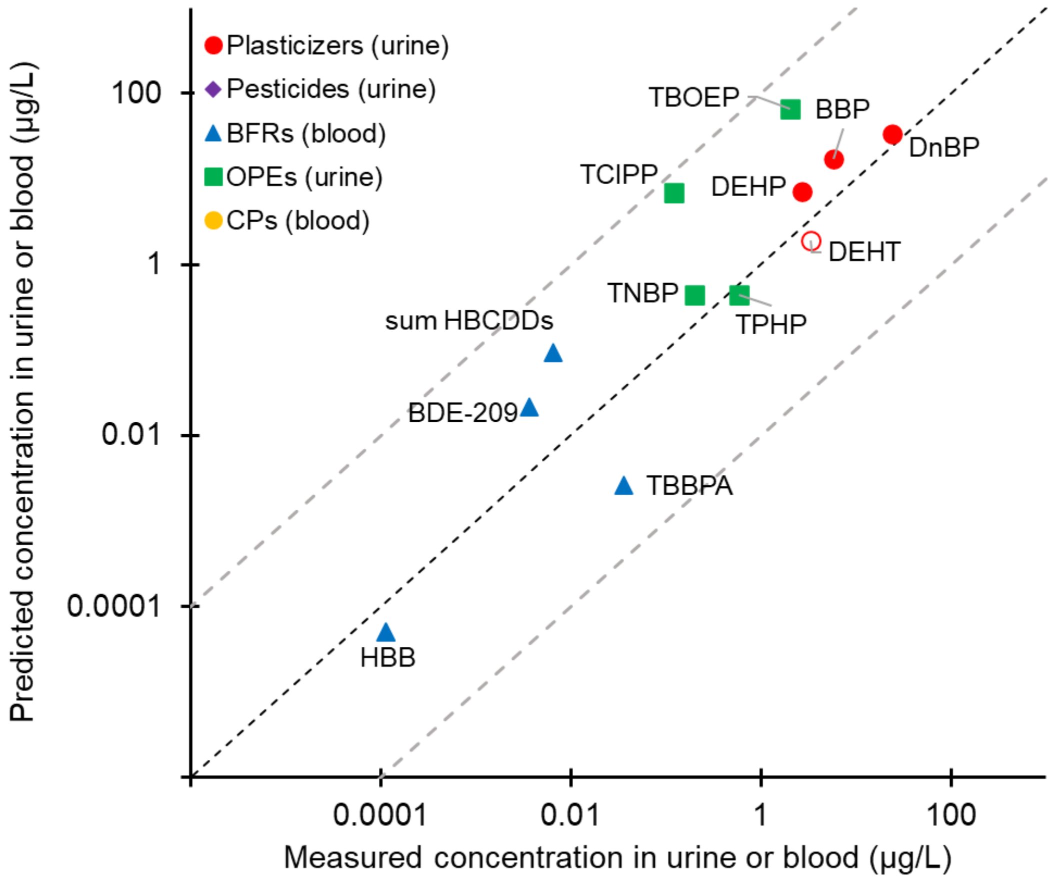

Figure S4: Comparison of RAIDAR-ICE-calculated concentrations in blood and urine to HBM data from the literature, assuming 3rd quartile dust concentrations from the highest contaminated locations measured in the SHINE project, and an adult dust ingestion rate of 22 mg/d. Dietary intake was not included in the calculation of DEHT metabolites in urine (empty marker). Black dashed lines represent agreement between measured and modeled values, and grey dashed lines show values in agreement within ± two orders of magnitude.

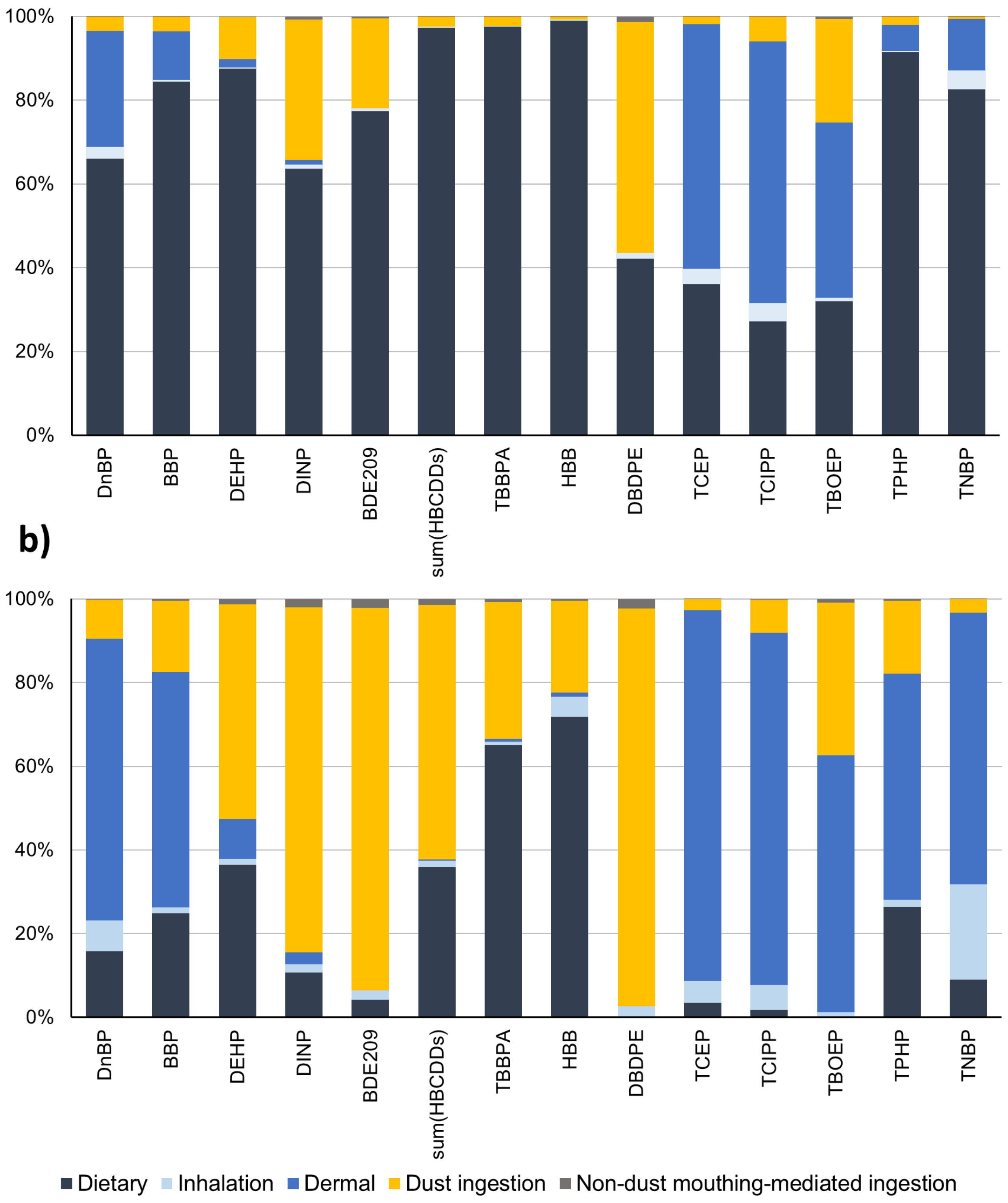

Figure S5: Relative contribution of different exposure pathways, assuming a) median concentrations in dust and a dust ingestion rate of 22 mg/day, and b) higher (3rd quartile) measured dust concentrations and a dust ingestion rate of 22 mg/day.

References

(1) Li, L.; Westgate, J. N.; Hughes, L.; Zhang, X.; Givehchi, B.; Toose, L.; Armitage, J. M.; Wania, F.; Egeghy, P.; Arnot, J. A. A Model for Risk-Based Screening and Prioritization of Human Exposure to Chemicals from Near-Field Sources. *Environ Sci Technol* **2018**, *52* (24), 14235-14244. DOI: 10.1021/acs.est.8b04059

(2) Weschler, C. J. Roles of the human occupant in indoor chemistry. *Indoor Air* **2016**, *26* (1), 6-24. DOI: https://doi.org/10.1111/ina.12185.

(3) Li, L.; Hughes, L.; Arnot, J. A. Addressing uncertainty in mouthing-mediated ingestion of chemicals on indoor surfaces, objects, and dust. *Environ Int* **2021**, *146*, 106266. DOI: 10.1016/j.envint.2020.106266

(4) Wilson, R.; Jones-Otazo, H.; Petrovic, S.; Mitchell, I.; Bonvalot, Y.; Williams, D.; Richardson, G. M. Revisiting Dust and Soil Ingestion Rates Based on Hand-to-Mouth Transfer. *Human and Ecological Risk Assessment: An International Journal* **2013**, *19* (1), 158-188. DOI: 10.1080/10807039.2012.685807.

(5) Layton, D. W.; Beamer, P. I. Migration of Contaminated Soil and Airborne Particulates to Indoor Dust. *Environmental Science & Technology* **2009**, *43* (21), 8199-8205. DOI: 10.1021/es9003735.

(6) USEPA. *Exposure Factors Handbook Chapter 5 (Update): Soil and dust ingestion*; EPA/600/R-17/384F; U.S. EPA Office of Research and Development, Washington, DC, 2017.

(7) Giovanoulis, G.; Bui, T.; Xu, F.; Papadopoulou, E.; Padilla-Sanchez, J. A.; Covaci, A.; Haug, L. S.; Palm Cousins, A.; Magnér, J.; Cousins, I. T.; et al. Corrigendum to "Multi-pathway human exposure assessment of phthalate esters and DINCH" [Environ. Int. 112 (2018) 115-126]. *Environ Int* **2020**, *143*, 106071. DOI: 10.1016/j.envint.2020.106071.

(8) Fierens, T.; Standaert, A.; Cornelis, C.; Sioen, I.; De Henauw, S.; Willems, H.; Bellemans, M.; De Maeyer, M.; Van Holderbeke, M. A semi-probabilistic modelling approach for the estimation of dietary exposure to phthalates in the Belgian adult population. *Environ Int* **2014**, *73*, 117-127. DOI: 10.1016/j.envint.2014.07.017

(9) EFSA. The 2016 European Union report on pesticide residues in food. *EFSA Journal* **2018**, *16* (7), e05348. DOI: 10.2903/j.efsa.2018.5348.

(10) Chain, E. P. o. C. i. t. F. Scientific Opinion on Polybrominated Diphenyl Ethers (PBDEs) in Food. *EFSA Journal* **2011**, *9* (5), 2156. DOI: https://doi.org/10.2903/j.efsa.2011.2156.

(11) Abdallah, M. A.; Harrad, S. Tetrabromobisphenol-A, hexabromocyclododecane and its degradation products in UK human milk: relationship to external exposure. *Environ Int* **2011**, *37* (2), 443-448. DOI: 10.1016/j.envint.2010.11.008

(12) Chain, E. P. o. C. i. t. F. Scientific Opinion on Tetrabromobisphenol A (TBBPA) and its derivatives in food. *EFSA Journal* **2011**, *9* (12), 2477. DOI: https://doi.org/10.2903/j.efsa.2011.2477.

(13) Zacs, D.; Perkons, I.; Abdulajeva, E.; Pasecnaja, E.; Bartkiene, E.; Bartkevics, V. Polybrominated diphenyl ethers (PBDEs), hexabromocyclododecanes (HBCDD), dechlorane-related compounds (DRCs), and emerging brominated flame retardants (EBFRs) in foods: The levels, profiles, and dietary intake in Latvia. *Sci Total Environ* **2021**, *752*, 141996. DOI: 10.1016/j.scitotenv.2020.141996.

(14) Ma, Y.; Stubbings, W. A.; Abdallah, M. A.; Cline-Cole, R.; Harrad, S. Temporal trends in concentrations of brominated flame retardants in UK foodstuffs suggest active impacts of global phase-out of PBDEs and HBCDD. *Sci Total Environ* **2023**, *863*, 160956. DOI: 10.1016/j.scitotenv.2022.160956.

(15) Poma, G.; Sales, C.; Bruyland, B.; Christia, C.; Goscinny, S.; Van Loco, J.; Covaci, A. Occurrence of Organophosphorus Flame Retardants and Plasticizers (PFRs) in Belgian Foodstuffs and Estimation of the Dietary Exposure of the Adult Population. *Environ Sci Technol* **2018**, *52* (4), 2331-2338. DOI: 10.1021/acs.est.7b06395

(16) Gbadamosi, M. R.; Abdallah, M. A.; Harrad, S. Organophosphate esters in UK diet; exposure and risk assessment. *Sci Total Environ* **2022**, *849*, 158368. DOI: 10.1016/j.scitotenv.2022.158368.

(17) Yuan, B.; Strid, A.; Darnerud, P. O.; de Wit, C. A.; Nyström, J.; Bergman, Å. Chlorinated paraffins leaking from hand blenders can lead to significant human exposures. *Environment international* **2017**, *109*, 73-80. DOI: j.envint.2017.09.014.

(18) Wormuth, M.; Scheringer, M.; Vollenweider, M.; Hungerbuhler, K. What are the sources of exposure to eight frequently used phthalic acid esters in Europeans? *Risk Anal* **2006**, *26* (3), 803-824. DOI: 10.1111/j.1539-6924.2006.00770.x

(19) Koch, H. M.; Rüther, M.; Schütze, A.; Conrad, A.; Pälmke, C.; Apel, P.; Brüning, T.; Kolossa-Gehring, M. Phthalate metabolites in 24-h urine samples of the German Environmental Specimen Bank (ESB) from 1988 to 2015 and a comparison with US NHANES data from 1999 to 2012. *Int J Hyg Environ Health* **2017**, *220* (2 Pt A), 130-141. DOI: 10.1016/j.ijheh.2016.11.003.

(20) SMG. *Humaan Biomonitoringsprogramma 2012-2015 - Luik Referntiebiomonitoring Jongeren Samenvatting ResultatenRapport*; Flemish Center of Expertise on Environment and Health (Steunpunt Milieu en Gesondheid), 2016. https://www.milieu-en-gezondheid.be/sites/default/files/atoms/files/Samenvatting%20jongeren%20ref%20steunpunt%203.pdf (accessed 2022-07-25).

(21) SMG. *Steunpunt II Referentiebiomonitoring*. 2012. https://www.milieu-en-gezondheid.be/nl/steunpunt-ii-referentiebiomonitoring (accessed 2022 2022-07-25).

(22) Lessmann, F.; Kolossa-Gehring, M.; Apel, P.; Rüther, M.; Pälmke, C.; Harth, V.; Brüning, T.; Koch, H. M. German Environmental Specimen Bank: 24-hour urine samples from 1999 to 2017 reveal rapid increase in exposure to the para-phthalate plasticizer di(2-ethylhexyl) terephthalate (DEHTP). *Environ Int* **2019**, *132*, 105102. DOI: 10.1016/j.envint.2019.105102.

(23) Vogel, N.; Frederiksen, H.; Lange, R.; Jørgensen, N.; Koch, H. M.; Weber, T.; Andersson, A. M.; Kolossa-Gehring, M. Urinary excretion of phthalates and the substitutes DINCH and DEHTP in Danish young men and German young adults between 2000 and 2017 - A time trend analysis. *Int J Hyg Environ Health* **2023**, *248*, 114080. DOI: 10.1016/j.ijheh.2022.114080.

(24) Norén, E.; Lindh, C.; Rylander, L.; Glynn, A.; Axelsson, J.; Littorin, M.; Faniband, M.; Larsson, E.; Nielsen, C. Concentrations and temporal trends in pesticide biomarkers in urine of Swedish adolescents, 2000-2017. *J Expo Sci Environ Epidemiol* **2020**, *30* (4), 756-767. DOI: 10.1038/s41370-020-0212-8.

(25) Jörgen Magnér, J.; Petra Wallberg, P.; Jasmin Sandberg, J.; Cousins, A. P. *Human exposure to pesticides from food: a pilot study*; Svenska MIljöinstitutet, 2015. https://static.julinse.com/m/16faf02308e82367.pdf.

(26) Sahlström, L. M. O.; Sellström, U.; de Wit, C. A.; Lignell, S.; Darnerud, P. O. Brominated Flame Retardants in Matched Serum Samples from Swedish First-Time Mothers and Their Toddlers. *Environmental Science & Technology* **2014**, *48* (13), 7584-7592. DOI: 10.1021/es501139d.

(27) Tay, J. H.; Sellström, U.; Papadopoulou, E.; Padilla-Sánchez, J. A.; Haug, L. S.; de Wit, C. A. Serum concentrations of legacy and emerging halogenated flame retardants in a Norwegian cohort: Relationship to external exposure. *Environ Res* **2019**, *178*, 108731. DOI: 10.1016/j.envres.2019.108731.

(28) Bjermo, H.; Aune, M.; Cantillana, T.; Glynn, A.; Lind, P. M.; Ridefelt, P.; Darnerud, P. O. Serum levels of brominated flame retardants (BFRs: PBDE, HBCD) and influence of dietary factors in a population-based study on Swedish adults. *Chemosphere* **2017**, *167*, 485-491. DOI: 10.1016/j.chemosphere.2016.10.008.

(29) Thomas, G. O.; Wilkinson, M.; Hodson, S.; Jones, K. C. Organohalogen chemicals in human blood from the United Kingdom. *Environ Pollut* **2006**, *141* (1), 30-41. DOI: 10.1016/j.envpol.2005.08.027

(30) Roosens, L.; Abdallah, M. A.; Harrad, S.; Neels, H.; Covaci, A. Exposure to hexabromocyclododecanes (HBCDs) via dust ingestion, but not diet, correlates with concentrations in human serum: preliminary results. *Environ Health Perspect* **2009**, *117* (11), 1707-1712. DOI: 10.1289/ehp.0900869

(31) Cequier, E.; Marcé, R. M.; Becher, G.; Thomsen, C. Comparing human exposure to emerging and legacy flame retardants from the indoor environment and diet with concentrations measured in serum. *Environment international* **2015**, *74*, 54-59. DOI: https://doi.org/10.1016/j.envint.2014.10.003.

(32) Ålander, J.; Gyllenhammar, I.; Bergh, A.; Cantillana, T.; Lignell, S.; Ankarberg, E., M. *Concentrations of brominated flame retardants (HBB, PBEB, BTBPE, DBDPE, PBDEs and HBCD) in blood serum from firsttime mothers in Uppsala 1996-2017*; Livsmedelsverket, 2019. http://www.imm.ki.se/Datavard/Rapporter/Sakrapport%20BFR%202016-2017.pdf.

(33) Schindler, B. K.; Förster, K.; Angerer, J. Determination of human urinary organophosphate flame retardant metabolites by solid-phase extraction and gas chromatography-tandem mass spectrometry. *J Chromatogr B Analyt Technol Biomed Life Sci* **2009**, *877* (4), 375-381. DOI: 10.1016/j.jchromb.2008.12.030.

(34) Bastiaensen, M.; Gys, C.; Malarvannan, G.; Fotache, M.; Bombeke, J.; Ait Bamai, Y.; Araki, A.; Covaci, A. Short-term temporal variability of urinary biomarkers of organophosphate flame retardants and plasticizers. *Environ Int* **2021**, *146*, 106147. DOI: 10.1016/j.envint.2020.106147.

(35) Fromme, H.; Lahrz, T.; Kraft, M.; Fembacher, L.; Mach, C.; Dietrich, S.; Burkardt, R.; Volkel, W.; Goen, T. Organophosphate flame retardants and plasticizers in the air and dust in German daycare centers and human biomonitoring in visiting children (LUPE 3). *Environ Int* **2014**, *71*, 158-163. DOI: 10.1016/j.envint.2014.06.016

(36) Yuan, B.; Haug, L. S.; Tay, J. H.; Padilla-Sánchez, J. A.; Papadopoulou, E.; de Wit, C. A. Dietary Intake Contributed the Most to Chlorinated Paraffin Body Burden in a Norwegian Cohort. *Environ Sci Technol* **2022**, *56* (23), 17080-17089. DOI: 10.1021/acs.est.2c04998.

(37) *Estimation Programs Interface Suite™ for Microsoft® Windows, v 4.11*; U.S. Environmental Protection Agency: Washington D.C., USA, 2012.

(38) Bureau, E. C. *EU-RAR (European Union-Risk Assessment Report). Alkanes, C10-13, chloro. CAS No: 85535-84-8. EINECS No: 287-476-5.*; Institute for Health and Consumer Protection, 2008. https://echa.europa.eu/documents/10162/c157d3ab-0ba7-4915-8f30-96427de56f84.

(39) Bureau, E. C. *EU-RAR (European Union-Risk Assessment Report). Alkanes, C14-17, chloro (MCCP). Part II – human health. CAS No: 85535-85-9. EINECS No: 287-477-0.*; Institute for Health and Consumer Protection, 2011. http://publications.jrc.ec.europa.eu/repository/bitstream/JRC66049/lbna25202enn.pdf.

(40) David, R. M. Exposure to phthalate esters. *Environmental Health Perspectives* **2000**, *108* (10), A440-A440. DOI: doi:10.1289/ehp.108-a440a.

(41) Apel, P.; Rousselle, C.; Lange, R.; Sissoko, F.; Kolossa-Gehring, M.; Ougier, E. Human biomonitoring initiative (HBM4EU) - Strategy to derive human biomonitoring guidance values (HBM-GVs) for health risk assessment. *Int J Hyg Environ Health* **2020**, *230*, 113622. DOI: 10.1016/j.ijheh.2020.113622.

(42) Apel, P.; Angerer, J.; Wilhelm, M.; Kolossa-Gehring, M. New HBM values for emerging substances, inventory of reference and HBM values in force, and working principles of the German Human Biomonitoring Commission. *Int J Hyg Environ Health* **2017**, *220* (2 Pt A), 152-166. DOI: 10.1016/j.ijheh.2016.09.007

(43) Itoh, H.; Yoshida, K.; Masunaga, S. Quantitative identification of unknown exposure pathways of phthalates based on measuring their metabolites in human urine. *Environ Sci Technol* **2007**, *41* (13), 4542-4547. DOI: 10.1021/es062926y

(44) Koch, H. M.; Christensen, K. L.; Harth, V.; Lorber, M.; Bruning, T. Di-n-butyl phthalate (DnBP) and diisobutyl phthalate (DiBP) metabolism in a human volunteer after single oral doses. *Archives of toxicology* **2012**, *86* (12), 1829-1839. DOI: 10.1007/s00204-012-0908-1

(45) Koch, H. M.; Bolt, H. M.; Preuss, R.; Angerer, J. New metabolites of di(2-ethylhexyl)phthalate (DEHP) in human urine and serum after single oral doses of deuterium-labelled DEHP. *Archives of toxicology* **2005**, *79* (7), 367-376. DOI: 10.1007/s00204-004-0642-4

(46) Lessmann, F.; Correia-Sá, L.; Calhau, C.; Domingues, V. F.; Weiss, T.; Brüning, T.; Koch, H. M. Exposure to the plasticizer di(2-ethylhexyl) terephthalate (DEHTP) in Portuguese children – Urinary metabolite levels and estimated daily intakes. *Environment international* **2017**, *104*, 25-32. DOI: j.envint.2017.03.028.

(47) Koch, H. M.; Schutze, A.; Palmke, C.; Angerer, J.; Bruning, T. Metabolism of the plasticizer and phthalate substitute diisononyl-cyclohexane-1,2-dicarboxylate (DINCH((R))) in humans after single oral doses. *Archives of toxicology* **2013**, *87* (5), 799-806. DOI: 10.1007/s00204-012-0990-4

(48) Leng, G.; Koch, H. M.; Gries, W.; Schütze, A.; Langsch, A.; Brüning, T.; Otter, R. Urinary metabolite excretion after oral dosage of bis(2-propylheptyl) phthalate (DPHP) to five male volunteers--characterization of suitable biomarkers for human biomonitoring. *Toxicology letters* **2014**, *231* (2), 282-288. DOI: 10.1016/j.toxlet.2014.06.035

(49) Côté, J.; Bonvalot, Y.; Carrier, G.; Lapointe, C.; Fuhr, U.; Tomalik-Scharte, D.; Wachall, B.; Bouchard, M. A novel toxicokinetic modeling of cypermethrin and permethrin and their metabolites in humans for dose reconstruction from biomarker data. *PLoS One* **2014**, *9* (2), e88517. DOI: 10.1371/journal.pone.0088517

(50) Zhang, T.; Bai, X. Y.; Lu, S. Y.; Zhang, B.; Xie, L.; Zheng, H. C.; Jiang, Y. C.; Zhou, M. Z.; Zhou, Z. Q.; Song, S. M.; et al. Urinary metabolites of organophosphate flame retardants in China: Health risk from tris(2-chloroethyl) phosphate (TCEP) exposure. *Environ Int* **2018**, *121* (Pt 2), 1363-1371. DOI: 10.1016/j.envint.2018.11.006

(51) Wang, Y.; Li, W.; Martinez-Moral, M. P.; Sun, H.; Kannan, K. Metabolites of organophosphate esters in urine from the United States: Concentrations, temporal variability, and exposure assessment. *Environ Int* **2019**, *122*, 213-221. DOI: 10.1016/j.envint.2018.11.007

(52) Fujii, M.; Shinohara, N.; Lim, A.; Otake, T.; Kumagai, K.; Yanagisawa, Y. A study on emission of phthalate esters from plastic materials using a passive flux sampler. *Atmospheric Environment* **2003**, *37* (39), 5495-5504. DOI: https://doi.org/10.1016/j.atmosenv.2003.09.026.

(53) Xu, Y.; Liu, Z.; Park, J.; Clausen, P. A.; Benning, J. L.; Little, J. C. Measuring and predicting the emission rate of phthalate plasticizer from vinyl flooring in a specially-designed chamber. *Environ Sci Technol* **2012**, *46* (22), 12534-12541. DOI: 10.1021/es302319m.

(54) Kemmlein, S.; Hahn, O.; Jann, O. Emissions of organophosphate and brominated flame retardants from selected consumer products and building materials. *Atmospheric environment* **2003**, *37* (39-40), 5485-5493.

(55) Han, X.; Li, W.; Zhao, Y.; Zhuang, Y.; Jia, Q.; Guan, H.; Liu, J.; Wu, C. Organophosphate Esters in Building Materials from China: Levels, Sources, Emissions, and Preliminary Assessment of Human Exposure. *Environ Sci Technol* **2024**, *58* (5), 2434-2445. DOI: 10.1021/acs.est.3c08432.
